# Prioritising intervention areas for antimicrobial resistance in Nigeria's human and animal health sectors using a mixed-methods approach

**DOI:** 10.1016/j.onehlt.2025.101082

**Published:** 2025-05-21

**Authors:** Oche A. Awulu, Akinbowale Jenkins, Babatunde A. Balogun, Emelda E. Chukwu, Folorunso O. Fasina, Abiodun Egwuenu, Oyinlola O. Oduyebo, Tajudeen A. Bamidele, Simeon Cadmus, Mabel K. Aworh, Adewole A. Adekola, Andrew P. Desbois, Kennedy F. Chah, Lucy A. Brunton

**Affiliations:** a Veterinary Epidemiology, Economics and Public Health Group, Department of Pathobiology and Population Sciences, Royal Veterinary College, London, United Kingdom; b Arctech Innovation, London, United Kingdom; c Australian Institute of Health Service Management, University of Tasmania, Tasmania, Australia; d Center for Infectious Diseases Research, Microbiology Department, Nigerian Institute of Medical Research, Lagos, Nigeria; e Department of Veterinary Tropical Diseases, University of Pretoria, Pretoria, South Africa; f Nigeria Centre for Disease Control and Prevention, Abuja, Nigeria; g Department of Medical Microbiology & Parasitology, College of Medicine, Idi-Araba, Lagos, Nigeria; h Molecular Biology & Biotechnology Department, Nigerian Institute of Medical Research, Yaba, Lagos, Nigeria; i Department of Veterinary Public Health & Preventive Medicine and Centre for Control and Prevention of Zoonoses, University of Ibadan, Ibadan, Nigeria; j Nigerian Institute of Medical Research, Lagos, Nigeria; k Department of Biological and Forensic Sciences, Lloyd College of Health, Science, and Technology, Fayetteville State University, Fayetteville, NC, USA; l Harper and Keele Veterinary School, Keele University Campus, Keele, Staffordshire, United Kingdom; m Institute of Aquaculture, University of Stirling, Stirling, United Kingdom; n Department of Veterinary Microbiology and Immunology Faculty of Veterinary Medicine, Animal Health Antimicrobial Resistance Surveillance Sentinel Laboratory, Veterinary Teaching Hospital University of Nigeria Nsukka, Enugu, Nigeria; o Food and Agriculture Organization of the United Nations, Juba, South Sudan

**Keywords:** Antimicrobial resistance, Antimicrobial stewardship, One Health, Nigeria, Interventions

## Abstract

A One Health approach is essential to prioritise intervention areas to tackle antimicrobial resistance (AMR). This study aimed to identify and evaluate critical drivers and antimicrobial stewardship (AMS) challenges within Nigeria's human and animal health sectors.

Human (22) and animal (33) health professionals in Nigeria were asked via an online questionnaire to rank priority pathogens, AMS challenges, and AMR drivers identified by subject matter experts. Descriptive statistics and the Fisher's exact test were used to evaluate differences in rankings between sectors. Subsequently, a scoping literature review of peer-reviewed research and grey literature was conducted to evaluate the evidence supporting the rankings.

*Salmonella* spp. (28.5 %) and *Escherichia coli* (28.2 %) were selected as the top-ranked priority pathogens for AMR. The Fisher's exact test showed a significant association (*p* = 0.049) between profession and ranking of *Salmonella*, which was ranked higher by animal health professionals than their human health counterparts. Priority AMS challenges in both human and animal health sectors were “ease of access to over-the-counter antimicrobials” (14.9 % and 20.1 %, respectively) and “lack of awareness of AMR/AMS” (14.1 % and 20.4 %, respectively). “Lack of infection prevention and control (IPC)” (24.5 %) was the highest-ranked AMR driver across sectors. Differences were observed between the rankings human and animal health professionals gave to the challenge of access to veterinary expertise (*p* = 0.011), as medical doctors ranked this component higher than veterinarians. “Lack of IPC” (*p* = 0.022) and “environmental degradation” (*p* = 0.048) were ranked higher by medical doctors than veterinarians. Conversely, “unsanitary processes in the abattoir(s)” was ranked higher among veterinarians (*p* = 0.032). Of the 84 articles reviewed, 62 supported the rankings of AMS challenges in both sectors, while 24 captured relevant AMR drivers.

Our findings underscore the need for a One Health approach in Nigeria to improve AMS and curb AMR.

## 1 Background

Antimicrobials, including antibacterial, antiviral, antifungal, and antiparasitic agents, are substances that kill or inhibit the growth of microorganisms, serving in the management and treatment of infections caused by pathogens in humans, animals, and plants [1]. However, the beneficial use of these agents is threatened by the development of antimicrobial resistance (AMR), which renders the drugs ineffective in treating infections with resultant therapeutic failures, prolonged illnesses, disabilities, and even death [1]. Globally, AMR severely threatens human, animal, and ecosystem health, making it an important One Health challenge. It has been called a ‘silent pandemic’, with an estimated burden higher than those of human immunodeficiency virus or malaria [2]. On a global scale, AMR is estimated to have caused over 4.95 million deaths in 2019 [3] and if the trends persist, is projected to result in around 10 million deaths annually with a total economic cost of US$100 trillion by 2050 [4].

Although AMR is a global health problem, the burden is greater in low- and middle-income countries (LMICs) due to limited resources [5] and awareness. The inappropriate use of antimicrobials is a major driver of AMR, with use in livestock accounting for approximately two-thirds of global antimicrobial consumption [6]. Nigeria ranks among the top five countries projected to experience the highest increase in the utilisation of antimicrobials in food animals [7]. A recent survey revealed that over 70 % of farms in Nigeria use antimicrobials in their routine operations, especially for growth promotion [8]. Similarly, in the public health sector, antimicrobials are over-utilised [9] and there is over-prescription of antimicrobials in Nigerian hospitals, sometimes without prior relevant laboratory diagnosis [10]. Furthermore, only 30 % of the tertiary hospitals in Nigeria had AMS committees while only 10 % showed evidence of leadership commitment to AMS [11], thus highlighting the urgent need for a cohesive AMS intervention program [10,12,13].

Various factors, such as poor regulatory enforcement, poor prescription practices, high level of demands by patients, underutilisation of clinical microbiology and lack of access to diagnostics, and inadequate healthcare facilities, have been reported as major contributors to inappropriate antimicrobial use [[14], [15], [16]]. In addition, substandard and falsified antimicrobials remain prevalent in Sub-Saharan Africa [17]. Poor implementation of infection prevention and control (IPC) and biosecurity measures across health settings result in the over-reliance on antimicrobials to prevent infection spread [18,19]. Meanwhile, despite the widespread use of antimicrobials in the livestock sector, withdrawal periods are poorly observed, resulting in high levels of antimicrobial residues in animal products, which further drive AMR [14,20]. The release of untreated pharmaceutical wastewater has also been identified as a leading contributor to AMR in Nigeria [20]. Moreso, improper discharge of antimicrobials into the environment through effluent from various sources poses significant concerns, as over 30 % of antimicrobial doses are excreted in faeces and urine in their original form [21]. The release of untreated wastes containing antimicrobials creates a hotspot for the development and spread of AMR at the human, animal, and ecosystem interface.

Nigeria, like other countries, has taken steps to tackle the AMR challenge by establishing an AMR Technical Working Group and developing a 5-year National Action Plan (NAP) on AMR in 2017 [22], with a second 5-year NAP (NAP 2.0) published in 2024 [23]. Despite Nigeria's commitments to tackle AMR, accomplishing the set objectives is fraught with various challenges, as outlined in a multi-sectoral expert panel report [14]. Drivers of AMR highlighted in that report include inadequate AMR and AMS awareness across different sectors and the public, as well as poor AMS practices characterised by the ease of over-the-counter (OTC) purchase of antimicrobials, poor regulatory enforcement, limited laboratory facilities, and low levels of adherence to IPC and biosecurity measures [14]. A recent study showed that more than 71 % of doctors were unaware of AMS [10]. Similarly, veterinarians have demonstrated poor awareness, as shown in a national survey where only 21 % of veterinary respondents properly defined AMS, and almost 60 % were unfamiliar with the guidelines of the NAP [18]. Likewise, only 8.3 % of the general population showed a good understanding of AMR in a nationwide survey [9]. Gaps remain in the operationalisation of One Health interventions for AMR in Nigeria, highlighting the need for a strengthened One Health infrastructure to address this challenge [14].

Given these constraints, prioritising AMR drivers and AMS challenges through a One Health approach is essential to channel efforts toward the most critical areas. Thus, the aim of this study was to identify and prioritise AMR drivers and stewardship challenges within Nigeria's human and animal health sectors, using expert opinions and literature evidence, to make recommendations to support the implementation of Nigeria's NAP on AMR.

## 2 Methods

### 2.1 Approach

**Fig. 1:**
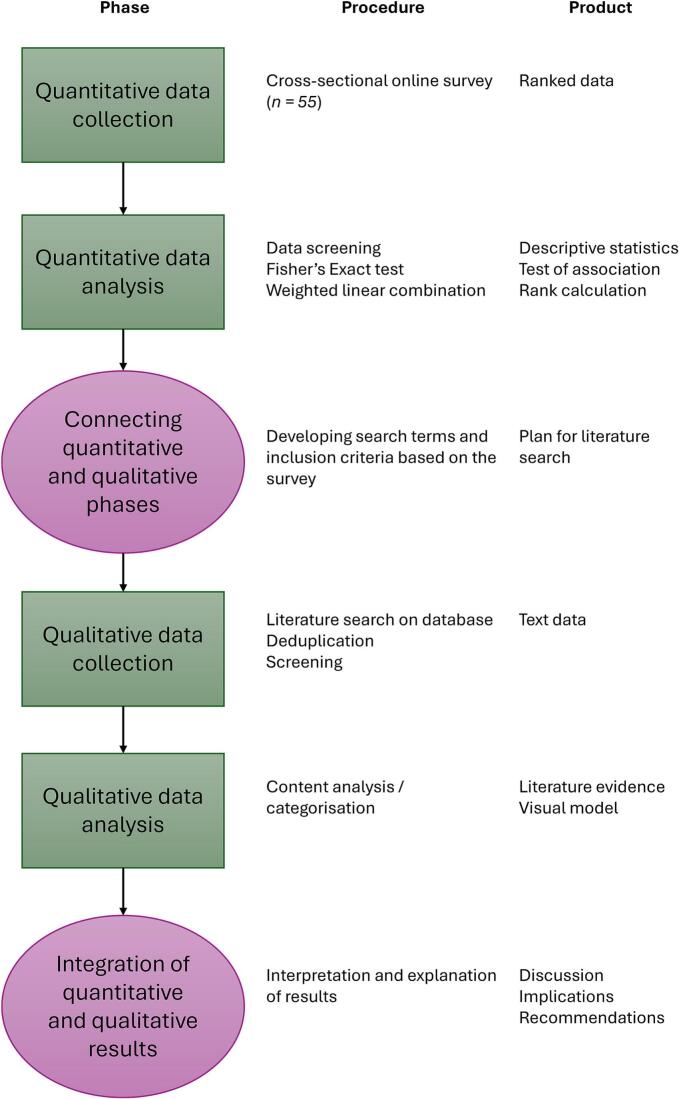
A visual model of the mixed-methods sequential explanatory design, adapted from [24].

This is a two-part study consisting of a field survey to rank preselected AMR drivers and AMS challenges followed by a scoping literature review to evaluate evidence supporting the rankings, as informed by an exploratory sequential study design [24,25]. A visual model of this study design, adapted from the procedure proposed by Ivankova et al. [24], is provided in Fig. 1.

### 2.2 Survey conception

In December 2022, the first meeting of the UK-Nigeria One Health AMR interest group was held with the aim of promoting collaborative AMR research between researchers in the UK and Nigeria. The nine attendees of the first meeting included academic researchers, government researchers and representatives from non-governmental public health organisations. One of the aims of the meeting was to discuss which AMR pathogens should be considered as priorities at the One Health interface, and which AMR drivers and AMS challenges exist for both the human and animal health sectors. During the meeting, attendees were split into two breakout groups and were requested to discuss these topics. Both groups fed back their discussions during a plenary session, where lists of priority One Health AMR pathogens, AMR drivers and AMS challenges for both sectors were agreed upon by consensus. The lists comprised of five pathogens, ten animal health AMS challenges, six human health AMS challenges, and five AMR drivers. Following the meeting, a short survey was developed using these lists as variables (i.e. themes) to be ranked in order of priority. Ten members of the wider UK-Nigeria One Health AMR interest group, consisting of four human health professionals (HHPs) and six animal health professionals (AHPs), pre-tested the survey.

### 2.3 Survey design

A cross-sectional survey was conducted over a 2-week period (15/06/2023 to 03/07/2023) using Microsoft Forms® (Supplementary file 1). It included a preliminary question on the respondent's profession and four ranking topics containing variables based on the lists developed during the initial meeting: priority pathogens (five options – 1 = highest priority, 5 = lowest priority), animal health AMS challenges (ten options – 1 = highest priority, 10 = lowest priority), human health AMS challenges (six options – 1 = highest priority, 6 = lowest priority), and AMR drivers (five options – 1 = highest priority, 5 = lowest priority). A final open question was included to capture any other drivers not identified during the survey conception.

### 2.4 Recruitment

Initial participants were recruited by a non-probability (snowball) sampling method through the professional contacts of the UK-Nigeria One Health AMR group who were encouraged to refer their professional colleagues through social media and email to the survey web link. The inclusion criterion was to be a practicing health professional in either the human or animal sector in Nigeria. Participants were required to respond to questions on both human and animal health sectors, regardless of their profession. The target number of respondents was 50, as this is the overall sample size recommended for a preliminary study of this kind by Sim & Lewis [26]. All responses that were at least 75 % complete were included in the data analysis.

### 2.5 Data analysis

The survey data were exported to Microsoft Excel® for cleaning and then imported into Statistical Package for Social Science (SPSS) version 26 for analysis. The distributions of the variables were checked and the frequencies, means and standard deviations (SD) determined. A weighted linear combination, as described by Roszkowska [27], was used to calculate the rank scores. Depending on the number of variables for ranking in each of the topics, an N-to-1 point scale was assigned, where N was the highest point assigned to the first rank followed by a decreasing order until 1. For example, where an item had five variables to rank, variables would be scored on a 5-to-1 point scale. The rank score for each variable was calculated as the sum of the products of the frequency and the assigned points (Eq. (1)).

(1) $$\text{Rank Score}=\sum \left(F_{1}*N\right)+\left(F_{2}*\left(N-1\right)\right)+\ldots \ldots \left(F_{Ø}*1\right)\;\left[27\right]$$

where: F represents the frequency of a variable in each rank (F_1_; first rank, F_2_; second rank, & F_Ø_; last rank). N represents the highest point assigned to the first rank, and 1 is assigned to the last rank.

The Fisher's exact test was used to test the association between the rankings assigned to individual variables and the two professions, with a *p*-value ≤0.05 considered as evidence of a significant association.

Responses on other AMR drivers (an open-ended question) were grouped into different categories using a qualitative content analysis method to describe textual data [28], where data are classified into a number of categories of similar meaning [29]. Categorized responses are presented in Supplementary file 2.

### 2.6 Evidence evaluation

A scoping review of the scientific literature related to AMS and AMR in Nigeria was conducted using specified search terms (Supplementary file 3) adapted across three databases (Scopus, Medline, and Web of Science), and the grey literature from government publications. The results were pooled and managed in Endnote version 20, following PRISMA guidelines [30]. The eligibility of studies for inclusion was assessed using the criteria in Table 1. The extracted data were categorized based on the variables in the survey and the degree to which they supported the rankings was evaluated.

**Table 1:** Inclusion and exclusion criteria for evidence evaluation.

Inclusion Criteria	• Peer-reviewed articles written in English. • Articles published from 01/01/2003 to 21/08/2024. • Articles focused on AMR drivers, stewardship challenges, and interventions in Nigeria's human or animal health sector. • Peer-reviewed articles with quantitative, qualitative, or mixed methods. • Non-peer-reviewed articles and grey literature, such as conference presentations and government reports.
Exclusion Criteria	• Duplicate articles. • Articles where the full text was not accessible. • Articles not relevant to study objectives, as determined by the primary author. • Secondary research, such as review articles, systematic reviews, and meta-analyses.

### 2.7 Ethical consideration

Ethical approval for the survey was received from the Social Science Research Ethical Review Board at the Royal Veterinary College (URN SR2023 - 0077) and the University of Nigeria Teaching Hospital Research Ethics Committee. Participants were required to provide informed written consent by indicating in their survey response that they had read and understood the information provided in the consent form and voluntarily agreed to participate before completing the survey (Supplementary file 1).

## 3 Results

### 3.1 Survey responses

Fifty-five survey responses were collected during the survey period and combined for analysis with the ten pilot responses from the UK-Nigeria One Health interest group workshop. The 55 responses included 22 (40 %) HHPs and 33 (60 %) AHPs. The majority of the respondents were actively involved in clinical practice in human and animal health sectors (81.8 % and 72.7 % respectively), across government and private settings (Table 2).

**Table 2:** Sector and areas of expertise of respondents.

Sector	N	Academia	Government agencies	Public health	Practice	
					Government	Private
Human Health	22	1 (4.5 %)	1 (4.5 %)	2 (9.1 %)	13 (59.1 %)	5 (22.7 %)
Animal Health	33	3 (9.1 %)	2 (6.1 %)	4 (12.1 %)	8 (24.2 %)	16 (48.5 %)

### 3.2 Ranking of priority pathogens

*Salmonella* spp. was ranked as the highest priority AMR pathogen (28.5 % of total rank score), followed closely by *E. coli* (28.2 %), while *Aeromonas hydrophila* (9.6 %) was ranked lowest (Table 3). The Fisher's exact test showed a significant association (*p* = 0.049) between profession and ranking of *Salmonella*.

**Table 3:** Scores and ranks of priority pathogens by profession (*N* = 54; missing =1).

Pathogen	Scores and ranks by profession				Total rank score	Percent (%)	Assigned rank	Fisher's exact test	P-value
	HHPs		AHPs						
	Rank score	Rank	Rank score	Rank					
*E. coli*	90	1	138	2	228	28.2	2	6.710	0.068
*Salmonella*	86	2	145	1	231	28.5	1	6.898	**0.049**
*Klebsiella*	68	3	84	3	152	18.8	3	4.711	0.293
*Enterococcus*	49	4	72	4	121	14.9	4	6.796	0.095
*Aeromonas*	37	5	41	5	78	9.6	5	2.974	0.223

Note: HHP = human health professional, AHP = animal health professional. Significant associations are in bold.

### 3.3 Animal health AMS challenges

The availability of (i.e. access to) antimicrobials OTC and lack of AMR awareness were the most prioritised stewardship challenges (14.9 % and 14.1 % of total rank score respectively). The lack of funding for relevant research was the least prioritised AMS challenge in the animal health sector (6.4 %). The Fisher's exact test showed a significant association (*p* = 0.011) between profession and ranking of lack of access to veterinary expertise with more HHPs ranking this higher than AHPs (Table 4).

**Table 4:** Scores and ranks of animal health AMS challenges by profession (*N* = 55).

Animal health AMS challenges	Scores and ranks by profession				Total rank score	Percent (%)	Assigned rank	Fisher's exact test	P-value
	HHPs		AHPs						
	Rank score	Rank	Rank score	Rank					
Availability of antimicrobial OTC	179	1	271	1	450	14.9	1	7.220	0.529
Lack of AMR/AMS awareness	179	1	248	2	427	14.1	2	10.219	0.195
Lack of laboratory facilities	149	3	213	4	362	12.0	3	10.076	0.318
Lack of regulation	122	5	214	3	336	11.1	4	8.382	0.513
Prescribers marketing antimicrobials	117	6	157	7	274	9.1	5	7.430	0.633
Lack of access to veterinary expertise	129	4	143	8	272	9.0	6	17.082	0.011*
Drug companies lobbying farmers	88	8	177	5	265	8.8	7	10.663	0.187
Lack of withdrawal period observation	85	9	160	6	245	8.1	8	9.851	0.345
Poor formulation/combination of antimicrobials	89	7	112	10	201	6.6	9	10.499	0.131
Lack of research funding	73	10	120	9	193	6.4	10	6.889	0.715

Note: HHP = human health professional, AHP = animal health professional. Significant associations are in bold.

### 3.4 Human health AMS challenges

The lack of awareness of AMR and ready availability of antimicrobials OTC were the most prioritised stewardship challenges (20.4 % and 20.1 % of the total rank score respectively). The disconnect between the Federal and State level on AMS coordination was the least prioritised stewardship challenge (12.8 %) (Table 5). No association was observed between profession and ranking of human health AMS challenges.

**Table 5:** Scores and ranks of human health AMS challenges by profession (*N* = 51; missing = 4).

Human health AMS challenges	Scores and ranks by profession				Total rank score	Percent (%)	Assigned rank	Fisher's exact test	P-value
	HHPs		AHPs						
	Rank score	Rank	Rank score	Rank					
Lack of awareness	92	1	126	2	218	20.4	1	9.252	0.077
Availability antimicrobial OTC	68	3	147	1	215	20.1	2	7.140	0.196
Hard to change practices & perceptions	72	2	102	4	174	16.3	3	3.263	0.700
Poor regulation of pharmaceutical industry	52	6	119	3	171	16.0	4	6.394	0.263
Lack of baseline information on AMR	59	4	97	5	156	14.6	5	1.721	0.922
Disconnect between Federal and State level^a^	56	5	81	6	137	12.8	6	1.923	0.919

a Nigeria operates a federal (national/first administrative level), state (subnational/second administrative level), and local levels of government. Note: HHP = human health professional, AHP = animal health professional.

### 3.5 AMR drivers

The lack of IPC and the absence of regulation that supports screening of antimicrobial residues were the most prioritised drivers of AMR (24.5 % and 23.2 % of the total rank score respectively). Environmental degradation was the least prioritised driver of AMR (15.1 %). The Fisher's exact test showed a significant association between profession and rankings for AMR drivers, like lack of IPC (*p* = 0.022), unsanitary processes in the abattoir(s) (*p* = 0.032), and environmental degradation (*p* = 0.048). HHPs ranked the lack of IPC and environmental degradation higher than AHPs. Conversely, unsanitary processes in the abattoir(s) as a driver were ranked higher among AHPs (Table 6).

**Table 6:** Scores and of ranks of AMR drivers by profession (*N* = 52; missing = 3).

AMR drivers	Scores and ranks by profession				Total rank score	Percent (%)	Assigned rank	Fisher's exact test	P-value
	HHPs		AHPs						
	Rank score	Rank	Rank score	Rank					
Lack of IPC	87	1	104	2	191	24.5	1	10.739	**0.022**
Absence of regulation that supports antimicrobial residue screening	57	2	124	1	181	23.2	2	7.240	0.113
Spillover from pharmaceutical wastes	56	4	94	4	150	19.2	3	1.424	0.913
Unsanitary processes in abattoir	43	5	97	3	140	18.0	4	10.083	**0.032**
Environmental degradation	57	2	61	5	118	15.1	5	8.895	**0.048**

Note: HHP = human health professional, AHP = animal health professional. Significant associations are in bold.

### 3.6 Literature search

**Fig. 2:**
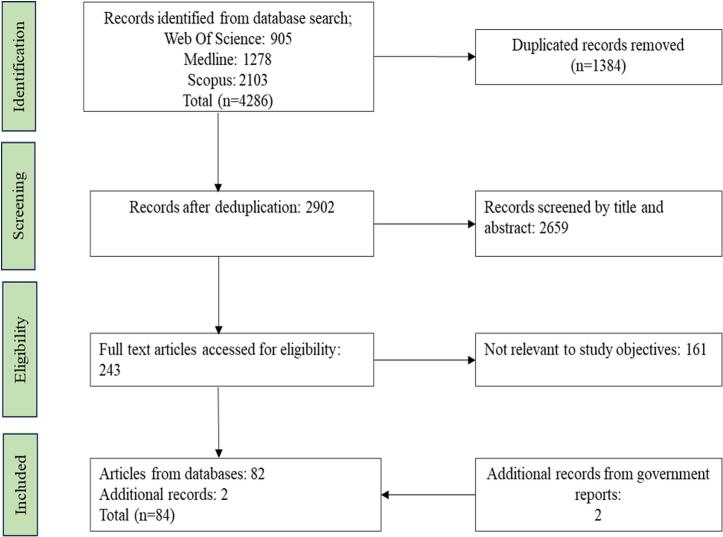
PRISMA flowchart of the literature search from databases.

The literature search from three databases yielded 4286 articles, which were checked for duplication and screened for eligibility and inclusion by the titles, abstracts, and full texts. Two government publications (29, 30) were also evaluated. Overall, 84 articles were included in the evaluation (Fig. 2).

#### 3.6.1 Evidence relating to challenges of antimicrobial stewardship

Of the 84 articles evaluated, 62 articles reflected the variables of the ranked stewardship challenges in both human and animal health sectors. A summary of the evidence provided by each article is provided in Supplementary file 4. The most commonly identified variables were poor AMR/AMS awareness and the availability of antimicrobials OTC, reflected 26 and 24 times respectively in the literature assessed. The evidence relating to challenges of antimicrobial stewardship is summarised below.

##### 3.6.1.1 Poor awareness of AMR and AMS

Despite being one of the key pillars of Nigeria's NAP, poor awareness on AMR and AMS is extensively documented among various stakeholders, including human health professionals [10,14,20,31,32], animal health professionals [14,18,33,34], farmers [[35], [36], [37], [38], [39], [40], [41], [42], [43]], the general public [9], and even medical science students [[44], [45], [46], [47]]. Consequently, the review of school curricula and the integration of AMR, IPC, and AMS into educational and professional training frameworks were identified as strategic interventions in Nigeria's National Action Plan on Antimicrobial Resistance [22]. Still, other studies that revealed appreciable AMR awareness reported that such enhanced awareness did not translate into prudent antimicrobial use [19,35,40,[48], [49], [50]]. Non-judicious use of antimicrobials in the face of increased AMR awareness was attributed to poor economic, infrastructural, and sociocultural constraints [19].

##### 3.6.1.2 Availability of antimicrobials OTC

The availability and ease of access to antimicrobials OTC was reported as an important stewardship challenge in 24 reviewed articles, including 8 in the human sector [9,12,14,20,[51], [52], [53], [54]], and 16 in the animal health sector [8,18,33,[35], [36], [37], [38],[55], [56], [57], [58], [59], [60], [61], [62], [63]].

##### 3.6.1.3 Weak regulation and regulatory enforcement

The pharmaceutical sector in Nigeria is deemed less than ideal, marked by counterfeit drugs, weak regulatory enforcement, and poor inter-agency cooperation [20]. The Nigerian veterinary legislation prohibits unqualified individuals from administering drugs, but enforcement of this is very limited [61]. Studies have revealed that a large percentage of animal health professionals and farmers are unaware of regulatory guidelines on antibiotic use and prohibited antimicrobials for animal use [40,55,60,62,64,65]. The weak enforcement regulations on antimicrobial access and usage were reported to significantly influence antimicrobial misuse in livestock [36,37].

##### 3.6.1.4 Prescribers marketing antimicrobials/financial influences on prescription

Studies have shown that farmers often consult unlicensed drug marketers for antimicrobial prescriptions, while veterinarians have been reported to sell antimicrobials through ambulatory drug stock or veterinary drug shops [60,65]. Pharmaceutical companies may exert undue influence over health professionals through drug promotional activities, using incentives to motivate the prescription of medications driven by profits from medication sales [19,66,67].

##### 3.6.1.5 Insufficient laboratory facilities and veterinary expertise

Studies report a lack of both government and private-owned veterinary laboratories in Nigeria, and that those that do exist are often underutilised due to the high service costs of reagents and materials, poor awareness, and the geographical remoteness of these facilities [14,18,68]. As a result, animal health professionals often resort to non-confirmatory assessments (syndromic, clinical and pathological) and empirical administration of broad-spectrum antimicrobials [19]. The position above is complicated by a shortage of veterinary experts in Nigeria [19,37,52]; only 7688 registered veterinarians were documented in Nigeria in 2017 [20], and specialists with requisite skills and competencies are even fewer.

##### 3.6.1.6 Hard to change behaviours and perceptions

Peer-reviewed evidence suggests that physicians often prescribe antimicrobials as a precautionary cover against infections [31,[69], [70], [71]]. Often, antimicrobial overprescription among HHPs is driven by the pressure to boost patient satisfaction, maintain clientele, and increase income [14,72,73]. Patients, on the other hand, often purchase drugs without a prescription and fail to complete prescribed dosages or may store them for future use [9,49,53,74,75]. Antimicrobials are viewed by many as a cure for all types of infections, including viral illnesses [31,76]. It has been reported that a majority (76.6 %) of individuals in the public domain felt powerless and had no control in mitigating the spread of AMR [9].

##### 3.6.1.7 Widespread disregard for compliance with withdrawal periods

Non-compliance with antimicrobial withdrawal periods is a prevalent practice among livestock farmers in Nigeria [8,[37], [38], [39],^42^,^56^,^58^,^64^,^77^,^78^]. This practice is driven by poor understanding of the impact of antimicrobial residues among farmers [14,79]. Even where the level of understanding is relatively high, there are instances of non-compliance with these withdrawal periods due to the fear of economic loss [14,68]. This non-compliance extends to imported products of animal origin where antimicrobial residues have been detected [80], underscoring a regulatory gap in the screening of antimicrobial residues.

##### 3.6.1.8 Poor formulation/combination of antimicrobials

In the animal health sector, antimicrobials are sometimes combined in a cocktail often called “com-biotics”, especially for growth promotion and chemo-prophylactic reasons [55,68,[81], [82], [83]].

##### 3.6.1.9 Inadequate funding, limited baseline information on AMR, and disconnect between Federal and State level

AMR research and development is poorly prioritised in Nigeria, due to limited government funding [84]. Similarly, there is inadequate surveillance and unreliable data on the pattern of AMR, due to limited national coordination and reporting systems for antimicrobial use and resistance [20,85]. The disconnect between Federal and State governments regarding AMS coordination results from the decentralized healthcare responsibilities outlined in the National Health Policy. This autonomy results in varying priorities, policies, and resource allocation, as evidenced by the absence of formal IPC programs in many primary and secondary facilities operated by local and state governments compared to Federal government-operated tertiary health facilities [22,86].

**Fig. 3:**
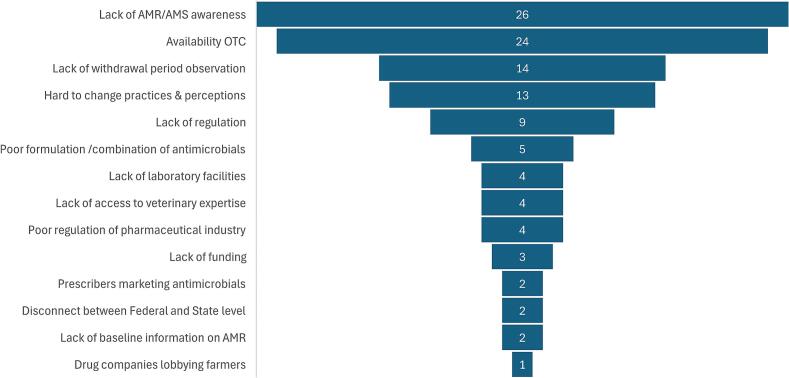
Funnel chart showing the distribution of evidence in the literature on AMS challenges in human and animal health sectors from 49 reviewed articles.

The findings from the literature review were in agreement with the top-ranked stewardship challenges in the survey. However, the lack of withdrawal period observation in food animals, which ranked lower in the survey, was abundantly captured in the literature review (Fig. 3 and Table 7).

**Table 7:** A comparison of the rankings of AMS challenges in animal and human health sectors, and supporting literature evidence.

Stewardship challenge	Animal health sector rank	Human health sector rank	Number of literature evidence
Availability OTC	1	2	24
Lack of AMR/AMS awareness	2	1	26
Lack of withdrawal period observation	8	–	14
Lack of regulation	4	–	9
Hard to change practices & perceptions	–	3	13
Poor formulation/combination of antimicrobials	9	–	5
Lack of laboratory facilities	3	–	4
Lack of access to veterinary expertise	6	–	4
Poor regulation of pharmaceutical industry	–	4	4
Lack of funding	10	–	3
Prescribers marketing antimicrobials	5	–	2
Disconnect between Federal and State level	–	6	2
Drug companies lobbying farmers	7	–	1
Lack of baseline information on AMR	–	5	2

#### 3.6.2 Evidence relating to AMR drivers

Twenty-four of the 84 evaluated articles captured themes of AMR drivers, with poor IPC mentioned in nine articles. The spillover of pharmaceutical wastes and unsanitary processes in the abattoir(s) were captured six times each, while there was no literature identified for the absence of regulation regarding antimicrobial residue screening (Table 8).

**Table 8:** A comparison of the rankings of AMR drivers and supporting literature evidence.

AMR drivers	Rank	Number of supporting articles
Lack of IPC	1	9
Absence of regulation that supports antimicrobial residue screening	2	–
Spillover from pharmaceutical wastes	3	6
Unsanitary processes in abattoir	4	6
Environmental degradation	5	3

Most Nigerian health institutions barely have functional IPC programs and facilities [20,86]. The difficulty in adhering to IPC measures is tied to negligence, inadequate training of health workers, poor infrastructure, and limited personal protective equipment (PPE) supply in healthcare settings [[87], [88], [89], [90], [91], [92]]. In the animal health sector, several animal health professionals admit to using antimicrobials as compensation for poor farm biosecurity [18]. Abattoirs and slaughterhouses have been identified as prominent hotspots for drug-resistant pathogens [[93], [94], [95], [96], [97]] due to unhygienic evisceration processes, improper waste treatment and disposal [98].

An analysis of Nigerian pharmaceutical companies revealed that nearly 80 % failed to remove pharmaceutical residues from wastewater discharged into the environment [99], and other studies have revealed high levels of resistance in bacterial isolates obtained from pharmaceutical effluents [[100], [101], [102], [103]]. The impact of AMR on the environment is poorly understood, as it is mainly viewed as a medical problem [20]. However, the indiscriminate disposal of manure has been identified as a major source of environmental degradation and pathogen spread in the environment [104,105]. This bears huge implications for the environment, as antimicrobial residues in the form of the parent compound or its metabolites are released into the environment following exposure to antimicrobials [20].

The ranking of AMR drivers in the survey is consistent with findings from the literature review, except for the absence of regulation regarding antimicrobial residue screening, for which no supporting evidence was found (Table 8).

## 4 Discussion

This mixed methods approach has allowed us to classify some of the most critical AMS challenges and AMR drivers in Nigeria. The findings from the examination of the literature generally supported the HHPs and AHPs prioritization of AMS challenges and AMR drivers established through the survey.

Although the selection of the priority AMR pathogens was guided by expert consensus within the UK-Nigeria One Health AMR Interest Group, their ranking in this survey is further validated by the recently released 2024 WHO Bacterial Priority Pathogens List (BPPL). Four of the five top-prioritised pathogens in this survey - *Klebsiella pneumoniae*, *Escherichia coli*, *Salmonella Typhi*, and *Enterococcus faecium* - are also listed among the WHO's globally prioritised pathogens [106]. *Salmonella* spp. and *E. coli* were considered by respondents to be the most important bacterial pathogens frequently demonstrating AMR in both human and animal health settings. Their relevance in Nigeria is well documented, with reports from the Nigeria Centre for Disease Control (NCDC) [20], and existing literature highlighting widespread resistance from the isolates of these pathogens [[107], [108], [109], [110], [111], [112]]. This evidence reinforces their role as significant enteric zoonotic pathogens and supports their prioritization in this survey.

However, in a recent zoonotic disease prioritization exercise in Nigeria, these two diseases were mid-ranked in terms of severity, epidemic potentials, associated burdens, ability to prevent and control, and their socio-economic impacts [113]. According to the WHO, members of the Enterobacteriaceae, including *E. coli* and *Klebsiella pneumoniae*, are categorized as critical AMR pathogens (priority 1), demonstrating resistance to carbapenem and third generation cephalosporins, while *Salmonella* spp. and *Enterococcus faecium* are categorized as priority 2 (high) pathogens demonstrating resistance against fluoroquinolone and vancomycin [114]. However, this only captures pathogens in human health settings, and similar evaluation and evidence generation in the animal health sector is warranted.

The low AMR awareness and ready availability of OTC antimicrobials were ranked in our survey as the most critical AMS challenges in both human and animal health sectors, and evidence obtained through the literature review supported this ranking. The observation that enhanced awareness does not always translate into prudent antimicrobial use [34,35,48,49] shows that AMR awareness and antimicrobial use often deviate from conventional expectations, buttressing the need for targeted behavioural change interventions. The fluctuating levels of AMR and AMS awareness may be because AMR awareness campaigns are mostly concentrated around the annual World AMR Awareness Week, rather than being delivered consistently [23]. In addition, there is a gap in AMR and AMS teaching in the training curricula of future healthcare professionals in Nigeria, which must be proactively addressed through revised curricula [45].

Easy access to antimicrobials OTC remains a predominant challenge limiting AMS in Nigeria, facilitated by socio-economic constraints, weak regulatory enforcement, poor coordination, and awareness among stakeholders in the drug use chain [14,20]. Poorly enforced regulations regarding access to antimicrobials OTC creates room for irrational antimicrobial prescriptions and sales, through the proliferation of under-regulated patent medicine vendors, unlicenced prescribers, and hawkers [20]. Similarly, the marketing of antimicrobials by some prescribers creates conflicts of interest, due to insufficient training [60], and financial incentives which potentially sway prescribers toward antimicrobial prescriptions even in cases where they are not required. To address these challenges, a whole-of-society approach with co-owned solutions involving all stakeholders must be implemented in access, coordination, monitoring, prescription and enforcement.

Some AMS challenges may have received a low ranking from respondents, not because they lack importance, but because of their perceptions and ingrained practices. Hard to change behaviours such as overprescription and self-medication, as well as poor perceptions, result in a lack of responsibility among stakeholders and the public to judiciously use antimicrobials [9,14,31]. Innovative ways to address these perceptions and practices must be engaged, and incorporation of behavioural science is likely to be key to achieving this. The lack of withdrawal period observation in food animals presents a noteworthy challenge to AMS, enhancing the propagation of AMR between animals and humans [20]. Despite the risk posed through the consumption of subtherapeutic levels of antimicrobial compounds, government regulation on screening of antimicrobial residues is markedly deficient, as Nigeria has no national program to monitor drug residues in food animal products [115]. However, the multisectoral stakeholders in AMR in Nigeria have developed a draft monitoring plan, and a programme does exist for monitoring residues in animal feeds [23].

Nigeria continues to grapple with limited infrastructure and human resources to tackle AMR. This is evidenced by the very limited number of government and privately-owned veterinary laboratories, which are often underutilised due to high costs, poor awareness, and location [14,18,68]. The lack of laboratory facilities hinders the precise identification of causative microorganisms and their susceptibility to specific antimicrobials to facilitate more targeted therapy. The lack of access to veterinary expertise also has a huge implication for animal health and AMS, as animal owners resort to quacks to accommodate the shortage of animal health professionals [19,60]. HHPs ranked this component higher than AHPs which could be attributed to their appreciation of the increasing trend in medical brain drain among healthcare workers, as less than half of registered doctors are reported to practice in the country [116]. Likewise, the differences observed in the ranking of AMR drivers between HHPs and AHPs, such as the lack of IPC, unsanitary processes in the abattoir(s), and environmental degradation, could be attributed to their differing perspectives. It is imperative that the government must focus on improved funding to support infrastructure and capacity development in these areas. Restructuring of certain areas of practice and incentivising good practice and dedication to duties, as well as co-financing with development partners, may assist in addressing these challenges.

To develop a holistic One Health approach to tackle AMR, Nigeria can leverage lessons learnt from high-income countries. For example, Denmark has achieved considerable success in curbing AMR through regulatory reforms to enhance the prudent use of antibiotics, improve IPC, and systematic surveillance of antibiotic consumption and resistance in both human and animal health settings [117]. Through restrictions on the use of antimicrobials crucial for human health in animals and the prohibited sales of antimicrobials for profit by professionals, Denmark has successfully reduced antibiotic consumption by over 29 % in the pig industry alone since 2010 [118]. In Africa, Kenya likewise developed a NAP for AMR, focusing on surveillance, stewardship, infection prevention and control, research, and multisectoral collaboration [119], and an aligned National Policy on AMR. Alongside the NAP and Policy, Kenya implemented guidelines to optimize and regulate antimicrobial use in both human and animal health settings, as well as a functional National IPC Programme to strengthen national and regional IPC governance [120]. Kenya has also made significant progress in incorporating AMR into educational curricula and training programs for healthcare workers. Such a comprehensive approach could support Nigeria's effort to curb AMR.

## 5 Limitations

The snowball sampling strategy utilised here, and the modest sample size could introduce bias, which may impact the generalizability and representativeness of this survey. Also, certain variables had limited counts, potentially influencing the robustness of the statistical comparisons. However, we have attempted to mitigate this by using the Fisher's Exact test instead of relying on the Chi-squared approximation. The literature search exposed a paucity of data to support an in-depth discussion of certain components, such as the lack of funding, lack of baseline AMR information, and environmental degradation, implying a research gap. Nevertheless, the insights from experts and literature remain valuable in identifying paramount concerns to inform AMR and AMS intervention strategies. While we are advocating a One Health approach to prioritising intervention areas for AMR in Nigeria, we acknowledge that this survey focused only on the human and animal health compartments. More work is required to identify and engage stakeholders within the environmental compartment to ensure that future interventions are truly holistic.

## 6 Conclusion

This study highlights the need to address AMR through a One Health approach in Nigeria. Expert opinion and literature evidence show that the lack of awareness and ready access to OTC antimicrobials are the most critical intersectoral AMS challenges in Nigeria. The identified association between profession and ranking of certain AMS challenges and AMR drivers could lead to disparities in decision-making and policy development. A collaborative One Health approach involving multi-level and multi-sectoral stakeholder engagement can address these disparities and provide valuable insights for policymakers and decision-makers to improve AMS and curb AMR spread.

## 7 Recommendations

As Nigeria renews her commitment to tackle AMR, the outcome of this study suggests that consideration should be given to improve AMR awareness, as well as the development and implementation of regulations that promote judicious antimicrobial access and use in both human and animal health settings. Therefore, our two primary recommendations are:

1. Address lack of awareness on AMR and failure of existing awareness campaigns by adopting a sustained approach using different mass media tools, and training of health professionals to improve public awareness and promote prudent antimicrobial usage. Strong integration of AMR and AMS modules within medical and veterinary school curricula is crucial to enhance knowledge and awareness among future healthcare professionals.
2. Strengthen regulations and regulatory enforcement within the country. Critical areas of regulatory reforms and implementation include regulated access to antimicrobials, screening for antimicrobial residues in animal products, and the treatment of pharmaceutical and agricultural effluents before disposal into the environment.

Further recommendations based on the evidence evaluated in this study would be to address the lack of IPC and lack of baseline data. Appropriate resources need to be allocated to enhance IPC and biosecurity in human and animal health facilities for IPC programmes, ample PPE supply, and proper waste management. Investments need to be made in research and development for AMR surveillance and innovative mitigation strategies. Finally, advocacy and stakeholder engagement for change needs to be increased, as desired actions depend on political will and effective communication.

## List of abbreviations

AHP: Animal health professional
AMR: Antimicrobial resistance
AMS: Antimicrobial stewardship
HHP: Human health professional
IPC: Infection prevention and control
LMIC: Low- and middle-income country
NAP: National action plan
NCDC: Nigeria Centre for Disease Control
OTC: Over the counter
PPE: Personal protective equipment
WHO: World Health Organization

## Authors' information

This study was undertaken by OAA in part fulfilment of the requirements for the degree of Master of Science in One Health: ecosystems, humans and animals at the Royal Veterinary College and the London School of Hygiene & Tropical Medicine, University of London.

## CRediT authorship contribution statement

**Oche A. Awulu:** Writing – original draft, Investigation, Formal analysis, Data curation. **Akinbowale Jenkins:** Writing – review & editing, Conceptualization. **Babatunde A. Balogun:** Writing – review & editing. **Emelda E. Chukwu:** Writing – original draft. **Folorunso O. Fasina:** Writing – review & editing. **Abiodun Egwuenu:** Writing – review & editing. **Oyinlola O. Oduyebo:** Writing – review & editing. **Tajudeen A. Bamidele:** Writing – review & editing. **Simeon Cadmus:** Writing – review & editing. **Mabel K. Aworh:** Writing – review & editing. **Adewole A. Adekola:** Writing – review & editing. **Andrew P. Desbois:** Writing – review & editing, Conceptualization. **Kennedy F. Chah:** Writing – review & editing, Project administration, Conceptualization. **Lucy A. Brunton:** Writing – review & editing, Supervision, Project administration, Methodology, Conceptualization.

## Consent for publication

Not applicable.

## Ethics approval and consent to participate

Ethical approval for the survey was received from the Social Science Research Ethical Review Board at the Royal Veterinary College (URN SR2023 - 0077) and the University of Nigeria Teaching Hospital Research Ethics Committee.

## Funding

This research did not receive any specific grant from funding agencies in the public, commercial, or not-for-profit sectors.

## Declaration of competing interest

The authors declare that they have no known competing financial interests or personal relationships that could have appeared to influence the work reported in this paper.

## Data Availability

All data generated or analysed during this study are included in this published article and its supplementary files.

## Appendix A Supplementary data

Supplementary material 1 Image 1

Supplementary material 2 Image 2

Supplementary material 3 Image 3

Supplementary material 4 Image 4
