# Supplementary material for: Prioritising intervention areas for antimicrobial resistance in Nigeria's human and animal health sectors using a mixed-methods approach
Source: One Health. 2025 May 21;20:101082. doi: 10.1016/j.onehlt.2025.101082 (PMC12166443; doi:10.1016/j.onehlt.2025.101082)
Supplement: Supplementary file 1 — Supplementary material 1 [file mmc1.pdf]

Supplementary material 1: Survey Questionnaire

UK-Nigeria
One Health AMR
Interest Group

Ranking AMR priorities in Nigeria

The following survey is designed to determine a ranking of the AMR problems and drivers identified at the inaugural UK-Nigeria One Health AMR interest group meeting. It should take approximately 7 minutes to complete.

* Required

1. **CONSENT INFORMATION**

   Title of Study: Prioritising Interventions for Antimicrobial Resistance (AMR) in Nigeria: A Data Analysis and Evidence Evaluation Study.

   Researcher(s):
   1. Dr. Lucy Brunton (Supervisor)
   2. Awulu Oche Abraham (Student)

Purpose of the study: The purpose of this study is to identify and evaluate the priority areas for intervention on antimicrobial resistance in Nigeria's veterinary and human health sectors.

Procedures: If you agree to participate, you will be asked to complete an online survey to capture your views on drivers of AMR in Nigeria. You will be asked to rank potential drivers in terms of their priority, and you will have the option to suggest drivers that may not be listed. The survey will take approximately 7 minutes to complete. Responses to the survey will be analysed at group level by the student researcher. We will also conduct a systematic literature review to evaluate the existing evidence supporting the prioritisation of the identified priority areas. This survey will be a part of an MSc dissertation, and is intended to contribute to existing knowledge on AMR in Nigeria. The results of this study may be published in scientific journals.

Risks and Benefits: There are no direct risks associated with participation in this study. However, some questions in the survey may be sensitive or personal in nature. You have the right to skip any question that you are uncomfortable answering. The benefits of this study include contributing to the development of targeted interventions in the highest priority areas to combat antimicrobial resistance in Nigeria.

Confidentiality: All information collected during the study will be kept strictly confidential.

Your identity will not be linked to any of your responses. Only the research team will have access to the data, which will be stored securely and destroyed once the study is completed. Ethical approval has been given by the Social Science Research Ethical Review Board at the Royal Veterinary College (URN SR2023 - 0077)

Voluntary participation: Participation in this study is completely voluntary. You may choose to withdraw from the study at any time without any negative consequences.

Consent: By agreeing to participate in this study, you acknowledge that you have read and understood this consent form and that you voluntarily agree to participate. If you have any questions or concerns about the study, you may contact the researcher at lbrunton@rvc.ac.uk, oawulu22@rvc.ac.uk.

I HEREBY CONSENT TO PARTICIPATE IN THIS SURVEY. *

- ○ Yes
- ○ No

2. I am over **18 years** old *

- ○ Yes
- ○ No

3. What institution or organisation are you affiliated with?

4. Please provide a few words to describe your expertise. *

5. Please rank the following pathogens exhibiting AMR by order of importance at the OH interface in Nigeria (top option is highest importance, bottom option is lowest importance)

- *Escherichia coli*
- *Salmonella* spp.
- *Klebsiella* spp.
- *Aeromonas hydrophila*
- *Enterococcus* spp.

6. Please rank the following problems impacting effective antimicrobial stewardship in the Nigerian animal production industry by order of importance (top option is highest importance, bottom option is lowest importance)

- Lack of awareness
- Lack of laboratory facilities for culture and sensitivity testing
- Availability of over-the-counter antimicrobials (human and animal)
- Lack of access to veterinary expertise
- Drug companies lobbying farmers
- Prescribers are those marketing drugs
- Poor formulations / combinations e.g. colistin Lack of withdrawal period observation
- Lack of regulations and regulatory enforcement
- Lack of funding for relevant research to back the need for AMR stewardship

7. Please rank the following problems impacting effective antimicrobial stewardship in the Nigerian health sector by order of importance (top option is highest importance, bottom option is lowest importance)

- Lack of awareness of AMR / AMS
- Hard to change existing behaviours and cultural perceptions Disconnect between Federal and State level
- Availability of over the counter drugs, particularly antibiotics Lack of baseline information about impact of AMR
- Poor regulation of pharmaceutical industry

8. Please rank the following drivers of AMR spread in Nigeria by order of importance (top option is highest importance, bottom option is lowest importance)

- Lack of infection prevention and control Environmental degradation
- Spillover of antimicrobials from drug production/waste or food crops
- Unsanitary processes in the abattoirs
- Absence of regulation that supports screening of antimicrobial residue in the food chain i.e. meat/fish/milk

9. Please list any other drivers of AMR spread in Nigeria that you think should be considered.
