# Supplementary material for: Prioritising intervention areas for antimicrobial resistance in Nigeria's human and animal health sectors using a mixed-methods approach
Source: One Health. 2025 May 21;20:101082. doi: 10.1016/j.onehlt.2025.101082 (PMC12166443; doi:10.1016/j.onehlt.2025.101082)
Supplement: Supplementary file 2 — Supplementary material 2 [file mmc2.pdf]

Supplementary material 2: Other Drivers of AMR Spread in Nigeria

Twenty eight (51%) of 55 respondents responded to the open-ended question on other drivers of AMR spread in Nigeria. The results were grouped into different categories, as presented in the Table below.

Table A: Categories of different AMR drivers and the comments from respondents

AMR Drivers (Category)	Comments from respondents
Inappropriate Usage	• *"Use of antimicrobials as growth promoters and disease prevention, use of antimicrobials without laboratory confirmation of bacterial involvement and no sensitivity testing, use of leftover antibiotics by farmers."* **P1 (A)**<br>• *"Use of antibiotics for prophylaxis and as growth promoters"* **P6 (A)**<br>• *"Indiscriminate use of antibiotics"* **P17 (A)**<br>• *"Under dosing by individuals who sell and or buy antibiotics"* **P20 (H)**<br>• *"Self- Medication"* **P28 (H)**<br>• *"Poultry and Fish production is among the top leading areas of Antimicrobials resistance and most widely consumed in most population i.e eggs or fish as fish roll and other foods ingredients, if the area can be looked into very well it can help in curtailing AMR as drugs are abused."* **P46(A)**<br>• *"Drug abuse"* **P34 (H)**<br>• *"Drug abuse"* **P55 (A)**<br>• *"excessive self medication"* **P19 (H)**<br>• *"Indiscriminate use, access and anecdotal use of antimicrobial agents for things it is not meant for by farmers e.g. before transportation to abattoir and animal market, as growth promoters."* **P10 (A)**<br>• *"Strict use of antibiotics to number of days required following culture"* **P21 (H)**
Skill and resource Gap	• *"Lack of formal training and qualifications"* **P2 (A)**<br>• *"Lack of effective cold chain"* **P2 (A)**
Poor Leadership/Coordination	• *"Non-existent or weak surveillance across sectors at national and sub-national levels"* **P3 (H)**<br>• *"WHO should be of help and individual or organization that are competent"* **P25 (H)**
Poor Practice	• *"Lack of biosecurity measures on farms"* **P6 (A)**<br>• *"Practitioners "covering up their inequities" with antimicrobials as an alternative to proper vaccination routine, biosecurity, IPC practices, proper aseptic conditions during surgery etc"* **P10 (A)**<br>• *"Not observing the withdrawal period recommended for antibiotic usage"* **P12 (A)**<br>• *Polypharmacy* **P34 (H)**
Regulation	• *"Absence of inter-state regulation in the transport of livestock and livestock-derived products such as meat, egg and milk."* **P9 (H)**

	• *Lack of incentives for farmers to not use antimicrobials, e.g. by insufficient differentiation of products in the market* **P2 (A)**<br>• *"Lack of farm registration process"* **P2 (A)**<br>• *"Poor regulation of Antibiotics"* **P13 (A)**<br>• *"Regulations of drugs shops"* **P18 (H)**<br>• *"Poor pharmaceutical controls"* **P37 (A)**<br>• *"Veterinary stores should also be regulated to sale only Antimicrobials and other biologicals strictly on prescription."* **P 46 (A)**
Environmental Degradation	• *"Disposal-related issues of antimicrobials (fostering environmental contamination and low-dose therapeutic levels in the environment - this is an issue with farms clearing out drinking water with antimicrobials added)"* **P10 (A)**<br>• *"Water and air pollution"* **P7 (A)**
Knowledge Gap & Attitude	• *"Poor motivation to participate as an AMR Steward"* **P11 (A)**<br>• *"Ignorance"* **P30 (H)**<br>• *"Poor attitude of drugs companies which focus on sales rather than the societal well-being"* **P48 (A)**<br>• *"Professional ethics and antimicrobial stewardship among veterinarians (disconnect between knowledge and what entails in their practice)"* **P10 (A)**
OTC Access	• *"Over the counter prescription"* **P16 (A)**<br>• *"Over the counter use of antibiotics"* **P19 (H)**<br>• *"Lack of control in prescriptions."* **P27(H)**<br>• *"Easy access to antimicrobial"* **P34(H)**<br>• *"Easy Access to Antimicrobial drugs without prescription"***P39(A)**
Quackery	• *"Abuse of antimicrobial by quacks"* **P17 (A)**<br>• *"Quackery in the veterinary and animal production sector (indiscriminate use of antibiotics by those poorly trained to understand the concept of antimicrobial use)"* **P10 (A)**<br>• *"Abolition of quacks"* **P18 (H)**<br>• *"Presence of quarks that disguises to be certified health professionals"* **P36 (A)**<br>• *"A lot quackery"* **P44 (A)**
Poor Laboratory Facilities	• *"Lack of back up evidence/indication for antimicrobial"* **P 34(H)**<br>• *""Broad spectrum" treatment without proper laboratory confirmation of the diagnosis in livestock and animal management. For instance, some dog breeders, paravets and some poorly trained animal handlers give a combination of oxytet, berenil, worming tablets, and multivitamins every time they notice signs of inappetence or ill-health in their dogs to cover for almost all possible aetiologies"* **P 10 (A)**

KEY: P= Participant; A=Animal health professionals; H=Human health professionals
