# Supplementary material for: Prioritising intervention areas for antimicrobial resistance in Nigeria's human and animal health sectors using a mixed-methods approach
Source: One Health. 2025 May 21;20:101082. doi: 10.1016/j.onehlt.2025.101082 (PMC12166443; doi:10.1016/j.onehlt.2025.101082)
Supplement: Supplementary file 3 — Supplementary material 3 [file mmc3.pdf]

Supplementary material 3: Search terms and results

SEARCH TERMS

Antimicrobial resistan* OR Antibiotic resistan* OR Drug resistan* OR multi-drug resistan* OR AMR OR AMR awareness OR infection prevention and control OR biosecurity OR environmental degradation OR Antimicrobial prescri* OR Antibiotic prescri* OR Antimicrobial access OR Antibiotic access OR Antimicrobial disposal OR Antibiotic disposal OR Antimicrobial stewardship* OR Antibiotic stewardship* OR Antimicrobial sensitivity test* OR Antibiotic sensitivity test* OR Antimicrobial withdrawal OR Antibiotic withdrawal OR growth promot* OR slaughterhouse hygiene OR abattoir hygiene AND Nigeria

RESULTS

1. SCOPUS

(TITLE-ABS-KEY ("Antimicrobial resistan*") OR TITLE-ABS-KEY ("Antibiotic resistan*") OR TITLE-ABS-KEY ("Drug resistan*") OR TITLE-ABS-KEY ("Multi-drug resistan* ") OR TITLE-ABS-KEY (amr) OR TITLE-ABS-KEY ("AMR awareness") OR TITLE-ABS-KEY ("Infection prevention and control") OR TITLE-ABS-KEY (biosecurity) OR TITLE-ABS-KEY ("environmental degradation") OR TITLE-ABS-KEY ("antimicrobial prescri*") OR TITLE-ABSKEY ("antibiotic prescri*") OR TITLE-ABS-KEY ("antimicrobial access") OR TITLE-ABS-KEY ("antibiotic access") OR TITLE-ABS-KEY ("antimicrobial disposal") OR TITLE-ABS-KEY ("antibiotic disposal") OR TITLE-ABS-KEY ("antimicrobial stewardship*") OR TITLE-ABS-KEY ("antibiotic stewardship*") OR TITLE-ABS-KEY ("antimicrobial sensitivity test*") OR TITLE-ABS-KEY ("antibiotic sensitivity test*") OR TITLE-ABS-KEY ("antimicrobial withdrawal") OR TITLE-ABS-KEY ("antibiotic withdrawal") OR TITLE-ABS-KEY ("growth promot*") OR TITLE-ABS-KEY ("abattoir hygiene") OR TITLE-ABS-KEY ("slaughterhouse hygiene") AND TITLE-ABS-KEY (nigeria)) AND PUBYEAR > 2002 AND PUBYEAR > 2002

RESULT: 2103

2. WEB OF SCIENCE

https://www.webofscience.com/wos/woscc/summary/e4635db2-b49c-48cb-b185-

6c8ee0a9271d-91852116/relevance/1

RESULT: 905

3. MEDLINE

1. ((Antimicrobial or antibiotic or drug or multi-drug) adj1 (resistan* or residue* or stewardship* or prescri* or access or disposal or sensitivity test* or withdrawal or awareness)).mp 455815

2. (AMR or (infection prevention and control) or biosecurity or environmental degradation or growth promot*).mp. 43518

3. ((abattoir or slaughterhouse) adj1 hygiene).mp. 13

4. Nigeria.mp. or Nigeria/ 45629

5. 1 or 2 or 3 491345

6. 4 and 5 1510

7. limit 6 to yr="2003 -Current" 1278

RESULT: 1278

TOTAL (SCOPUS + WEB OF SCIENCE + MEDLINE) = 4007
