# Supplementary material for: Prioritising intervention areas for antimicrobial resistance in Nigeria's human and animal health sectors using a mixed-methods approach
Source: One Health. 2025 May 21;20:101082. doi: 10.1016/j.onehlt.2025.101082 (PMC12166443; doi:10.1016/j.onehlt.2025.101082)
Supplement: Supplementary file 4 — Supplementary material 4 [file mmc4.pdf]

Supplementary material 4: Summary of the literature assessed

AMS CHALLENGES IN ANIMAL AND HUMAN SECTORS

AUTHOR/YEAR	TITLE	SECTOR	FINDINGS/OBSERVATIONS
**Availability of antimicrobials OTC**			
1. (Ezechukwu *et al.*, 2005)	Drug treatment of common childhood symptoms in Nnewi: what mothers do?	H	52.6% of mothers bypassed proper medical supervision, to administer antimicrobials and other medications to their children from drug stores.
2. (Esimone *et al.*, 2007)	Utilization of antimicrobial agents with and without prescription by out-patients in selected pharmacies in South-eastern Nigeria.	A	57.8% of out-patients in southeastern Nigeria obtained antimicrobials without prescription, for self-medication from community pharmacies.
3. (Omeiza *et al.*, 2012)	Response of Nigerian farmers to a questionnaire on chloramphenicol application in commercial layers.	A	Although chloramphenicol is prohibited, 34.3% of farmers purchased both human and veterinary preparations for use in commercial laying farms.
4. (Adesokan *et al.*, 2015)	Pattern of antimicrobial usage in livestock animals in south-western Nigeria: The need for alternative plans.	A	Antibiotics are widely available over the counter and purchased by livestock farmers without veterinary prescription and supervision in the study area.
5. (NCDC, 2017)	Antimicrobial Use and Resistance in Nigeria Situation Analysis and Recommendations.	A&H	The Nigerian legislation mandates that antimicrobials should only be dispensed with prescription. However, challenges such as shortage of licensed prescribers, proliferation of under-regulated patent medicine vendors and hawkers in others, contribute to the crisis of irrational drug use. Drug misuse extends to the agricultural sector where antimicrobials are liberally used therapeutically and for growth promotion.

6. (Ojo *et al*., 2017).	Activities and influence of veterinary drug marketers on antimicrobial usage in livestock production in Oyo and Kaduna States, Nigeria	A	45.4% of drug marketers sold antimicrobials without prescription to farmers.
7. (Alhaji & Isola, 2018)	Antimicrobial usage by pastoralists in food animals in North-central Nigeria: The associated socio-cultural drivers for antimicrobials misuse and public health implications.	A	58.3% and 65.4% of pastoralist practiced self-prescription and self-administration of antimicrobials. Drug shops (59.6%) and animal hawkers (34.9%) were the major source of antimicrobial purchase.
8. (Okpara *et al*., 2018)	Antimicrobial usage and presence of extended-spectrum β-lactamase-producing Enterobacteriaceae in animal-rearing households of selected rural and peri-urban communities.	A&H	69.4% of households indicated that they purchased antimicrobials for use in humans and animals without prescription from a physician.
9. (Alhaji *et al*., 2019)	Survey on antimicrobial usage in local dairy cows in North-central Nigeria: Drivers for misuse and public health threats.	A	Farmers purchased antimicrobials used on cows from veterinary drug shops (34.9%), human drug shops (10.7%), and drug hawkers (54.4%).
10. (Abubakar & Tangiisuran, 2020)	Knowledge and practices of community pharmacists towards non-prescription dispensing of antibiotics in Northern Nigeria.	H	39.7% of community pharmacists admitted to dispensing antibiotics to buyers without a prescription.
11. (Adekanye *et al*., 2020).	Knowledge, Attitudes and Practices of Veterinarians Towards Antimicrobial Resistance and Stewardship in Nigeria.	A	60% of veterinarians reported that animal owners usually purchased and initiated antibiotic treatment without veterinary supervision.
12. (Adelowo & Okunlola, 2020)	Field assessment of antibiotic use in fish farms in Southwestern Nigeria.	A	Fish farmers sourced antimicrobials from veterinary stores (59%) and human medicine stores (15%).
13. (Al-Mustapha *et al*., 2020).	Risk Perceptions of Antibiotic Usage and Resistance: A Cross-Sectional Survey of Poultry Farmers in Kwara State, Nigeria.	A	51.2% of farmers easily obtained antibiotics without prescription from a veterinarian.

14. (Chukwu *et al*., 2020)	A national survey of public awareness of antimicrobial resistance in Nigeria.	H	Most respondents (members of the public) purchased antibiotics from pharmacy store (70.3%), chemist (14.5%), and hawkers (2.1%) with or without prescription
15. (Ojo *et al*., 2020).	Antimicrobial use and detection of cefotaxime-resistant Enterobacteriaceae in the pig production chain, Ogun State, Nigeria.	A	100% of pig farmers reported that they had ready access to antimicrobial and had never been asked to provide a prescription before purchase
16. (Achi *et al*., 2021)	Operationalising One Health in Nigeria: Reflections from a High-Level Expert Panel Discussion Commemorating the 2020 World Antibiotics Awareness Week.	A&H	Over the counter access extends beyond first or second-line antibiotics, to critically important antimicrobials, some of which are "hawked" on the streets in certain regions of the country
17. (Adebowale *et al*., 2021)	Biosecurity and Antimicrobial Use Practices in Live Bird Markets within Abeokuta Metropolis, Southwest, Nigeria: A Preliminary Survey.	A	90.0% of farmers self-administered antimicrobials to poultry and purchased drugs from veterinary shops or human medicine stores without veterinary prescriptions. antimicrobials were chosen according to previous experience, accessibility, and cost.
18. (Alhaji *et al*., 2021)	Antimicrobial use, residue and resistance dissemination in freshwater fish farms of north-central Nigeria: One health implications.	A	Fish farmers purchased antimicrobials from veterinary drug shops (62.7%), human drug shops (26.3%), and drug hawkers (while 11.0%) without prescription.
19. (Aworh *et al*., 2021)	Assessing knowledge, attitude, and practices of veterinarians towards antimicrobial use and stewardship as drivers of inappropriate use in Abuja, Nigeria.	A	Veterinarians reported that a wide range of antibiotics classes including tetracycline, sulphonamides, penicillin, chloramphenicol, aminoglycosides, cephalosporins, macrolides, colistin, and Fosfomycin were commonly sold without a prescription to consumers, especially poultry farmers.
20. (Bamidele *et al*., 2022)	Antimicrobial Usage in Smallholder Poultry Production in Nigeria.	A	Farmers easily sourced antimicrobials from veterinary stores (34.0%), local vendors (30.2%), human pharmacies/chemists (21.2%), and feedstores (14.6%).
21. (Chah *et al*., 2022)	Knowledge and practices regarding antibiotic use among small-scale poultry farmers in Enugu State, Nigeria.	A	25% of farmers reportedly agreed to purchase antibiotics without veterinary prescription, while 80% agreed to obtain antibiotics prescription from another farmer.

22. (Alhaji *et al*., 2023)	Antimicrobial usage and associated residues and resistance emergence in smallholder beef cattle production systems in Nigeria: A One Health challenge.	A	74% of poultry farmers bought antibiotics without prescription from poultry drug stores, while the rest (26%) obtained theirs from private and government veterinary outlets.
23. (Busari et al., 2024)	Evaluation of antibiotic use and analysis of ciprofloxacin and gentamicin residue in fish samples from farms in Lagos, Nigeria	A	Over 40% of fish farmers in this study self-administered antibiotics in aquaculture for prophylactic purposes. Over 72% of farmers obtained antibiotics from local vendors.
24. (Amorha *et al.* 2024)	Assessment of Community Pharmacists' Knowledge, Perception and Practice Regarding Antimicrobial Stewardship in Ebonyi State, Southeastern Nigeria	H	40% of the respondents (community pharmacists) reported that they dispense antibiotics without prescription often/always.
**Lack of AMR/AMS awareness**			
1. (NCDC, 2017)	Antimicrobial Use and Resistance in Nigeria Situation Analysis and Recommendations.	A&H	Health professionals and the public are underinformed about AMS. There is also a gap in the teaching of AMR in the undergraduate curricula of healthcare professionals, thus requiring further review of the existing curricula.
2. (Alhaji *et al*., 2018)	Antimicrobials usage assessments in commercial poultry and local birds in North-central Nigeria: Associated pathways and factors for resistance emergence and spread.	A	Only 21.9% and 7.3% of farmers and bird keepers respectively, agreed that AMR pathogens in poultry can be transmitted to humans. Also, 46.4% of poultry farmers and 6.8% of bird keepers recognized antimicrobial misuse as administering an under-dosage.
3. (Alhaji & Isola, 2018)	Antimicrobial usage by pastoralists in food animals in North-central Nigeria: The associated socio-cultural drivers for antimicrobials misuse and public health implications.	A	67% of pastoralist surveyed did not know what AMR means. Only 28% of respondents demonstrated a correct understanding of AMR phenomenon. Only 8.1% of pastoralists understood antibiotic misuse as under-dosing, with 70.1% unaware of its meaning.
4. (Njoga *et al*., 2018)	Assessment of antimicrobial drug administration and antimicrobial residues in food animals in Enugu State, Nigeria.	A	74.3% of livestock farmers surveyed were unaware that imprudent use of antimicrobial drugs in food animals can enhance AMR development.

5. (Alex *et al*., 2019)	Knowledge of antibiotic use and resistance among students of a medical school in Nigeria.	H	98.4% of medical students demonstrated a good knowledge of antibiotic use and resistance, however, a significant proportion (44%) did not follow correct antibiotic use, with only 8.2% consistently consulting a doctor before using antibiotics.
6. (Alhaji *et al*., 2019)	Survey on antimicrobial usage in local dairy cows in North-central Nigeria: Drivers for misuse and public health threats.	A	51.6% and 60.4% of farmers did not know the consequences AMR in lactating cows and humans, respectively.
7. (Odetokun *et al*., 2019)	Knowledge of Antimicrobial Resistance among Veterinary Students and Their Personal Antibiotic Use Practices: A National Cross-Sectional Survey.	A	Less than half (42.3%) of Nigerian veterinary students surveyed were aware of the term "AMR" and only 13.1% of were aware of antimicrobial stewardship.
8. (Pearson & Chandler, 2019)	Knowing antimicrobial resistance in practice: a multi-country qualitative study with human and animal healthcare professionals.	A&H	Health professionals understood AMR and the link between antimicrobial use and its development but didn't consistently apply cautious antimicrobial practices. Surprisingly, awareness of AMR sometimes led to increased antimicrobial use due to economic, infrastructural, commercial, and social influences.
9. (Adekanye *et al*., 2020).	Knowledge, Attitudes and Practices of Veterinarians Towards Antimicrobial Resistance and Stewardship in Nigeria.	A	Only 36% of veterinarians in the survey had heard of the term antimicrobial stewardship. 59.8% of respondents were unaware of the guidelines provided by the Nigeria AMR National Action Plan.
10. (Chukwu *et al*., 2020)	A national survey of public awareness of antimicrobial resistance in Nigeria.	H	56.5% of the general public were familiar with the term "antibiotic resistance", only 8.3% had good knowledge of AMR.
11. (Fasina *et al*., 2020)	Knowledge, Attitudes, and Perceptions Associated with Antimicrobial Stewardship Among Veterinary Students: A Multi-Country Survey from Nigeria, South Africa, and Sudan.	A	68% of veterinary students from Nigeria, South Africa, and Sudan were confident on their knowledge regarding AMR. 71.6% of Nigerian students were confident of their AMR knowledge.
12. (Ojo *et al*., 2020).	Antimicrobial use and detection of cefotaxime resistant Enterobacteriaceae in the pig production chain, Ogun State, Nigeria.	A	65.0% farmers recognized pigs as potential carriers of bacteria transmissible to humans, but only 48.3% were aware that using antimicrobials could facilitate the development of antimicrobial-resistant bacteria.

13. (Achi *et al.*, 2021)	Operationalising One Health in Nigeria: Reflections from a High-Level Expert Panel Discussion Commemorating the 2020 World Antibiotics Awareness Week.	A&H	Public AMR awareness is poor and many professionals within the various One Health sectors are poorly aware of their role in ensuring antimicrobial stewardship
14. (Akande-Sholabi & Ajamu, 2021)	Antimicrobial stewardship: Assessment of knowledge, awareness of antimicrobial resistance and appropriate antibiotic use among healthcare students in a Nigerian University.	H	58.2% of healthcare students showed good knowledge of antimicrobial resistance, however, the knowledge did not reflect in antibiotic appropriate use as antibiotics were used in treatment/management of malaria (16.9 %), cough (16.3 %), and sore throat (15.9 %)
15. (Aworh *et al.*, 2021)	Assessing knowledge, attitude, and practices of veterinarians towards antimicrobial use and stewardship as drivers of inappropriate use in Abuja, Nigeria.	A	Only 18.1% of veterinarians in survey demonstrated good knowledge of AMR and stewardship. 48.6% had average knowledge, while 33.3% showed poor knowledge of the subject.
16. (Babatola *et al.*, 2021)	Addressing antimicrobial resistance in Nigerian hospitals: exploring physicians prescribing behaviour, knowledge, and perception of antimicrobial resistance and stewardship programs.	H	82.7% of physicians had good knowledge of AMR, but only 36.5% understood Antimicrobial Stewardship Programs, and only 28.2% knew about antibiotic stewardship.
17. (Chukwu *et al.*, 2021)	Antimicrobial resistance awareness and antibiotic prescribing behavior among healthcare workers in Nigeria: a national survey.	H	49.2% of healthcare professionals showed good AMR knowledge, while 47.2% had fair knowledge, and 3.6% had poor knowledge regarding AMR.
18. (Njoga *et al.*, 2021)	Antimicrobial drug usage pattern in poultry farms in Nigeria: Implications for food safety, public health and poultry disease management.	A	70.3% of poultry farmers were unaware that antibiotic misuse in poultry could result in AMR development in the food chain. Additionally, 67.9% of farmers were unaware that failure to observe withdrawal periods could lead to elevated levels of drug residues in poultry products, while 63.2% were unaware that consuming these residues could lead to health problems.

19. (Abdu-Aguye *et al*., 2022)	Investigating Knowledge of Antibiotics, Antimicrobial Resistance and Antimicrobial Stewardship Concepts Among Final Year Undergraduate Pharmacy Students in Northern Nigeria.	H	Majority (94.5%) of undergraduate pharmacy students surveyed were aware of antimicrobial resistance but only 48.8% of them knew what Antimicrobial Stewardship (AMS) entailed.
20. (Chah *et al*., 2022)	Knowledge and practices regarding antibiotic use among small-scale poultry farmers in Enugu State, Nigeria.	A	70.5 % of the farmers had good knowledge of AMR, however, all (100%) respondents in the survey used antibiotics for growth promotion and disease prevention in poultry. Study showed that farmers with good knowledge of AMR were more likely to misuse antibiotics than those with poor knowledge.
21. (Smith *et al*., 2022)	Antibiotic prescription practices amongst veterinarians in Nigeria.	A	46.5% of veterinarians were unaware of antibiotic prescription policy in Nigeria.
22. (Oloso *et al*., 2022)	Knowledge, Attitudes, and Risk Perception of Broiler Grow-Out Farmers on Antimicrobial Use and Resistance in Oyo State, Nigeria.	A	100% of poultry farmers in Oyo state knew antibiotics kill bacteria. Most (73.7%) believed giving antibiotics to poultry aids weight gain, and many (69.7%) thought antibiotics have no negative side effects.
23. (Idongesit et al., 2023)	Knowledge and perception of antimicrobial resistance and antimicrobial stewardship among healthcare students in Nigeria	H	Majority of students had good knowledge of AMR, as 94.3% viewed AMR as a worldwide problem. However, over half (60.8%) were not familiar with the term ‘antimicrobial stewardship’
24. (Huang & Eze, 2023)	Awareness and Knowledge of Antimicrobial Resistance, Antimicrobial Stewardship and Barriers to Implementing Antimicrobial Susceptibility Testing among Medical Laboratory Scientists in Nigeria: A Cross-Sectional Study	H	More than a quarter of respondents were unaware of AMR. More than two-thirds of respondents were unaware of “antibiotic stewardship” or “antimicrobial stewardship.”
25. (Yakubu et al., 2024)	Understanding the awareness of antimicrobial resistance amongst commercial poultry farmers in northwestern Nigeria	A	A significant number of the farmers have good knowledge of antimicrobial use (94.74 %), and the potential emergence of antimicrobial resistant bacteria due to irrational administration of

			antibiotics (83.0 %). However, a significant number of the farmers administer antibiotics even when the birds are not sick (40.49%),
26. (Olujide Ojo et al., 2024)	Antibiotics Use, Resistance and Self-medication Practices among Healthcare Workers in a Federal Teaching Hospital in Southwest, Nigeria	H	Although 82.2% of the respondents HHPs were aware of antibiotic resistance, only 39.4% of them had a positive attitude towards antibiotic use
**Lack of laboratory facilities for culture and sensitivity testing**			
1. (Pearson & Chandler, 2019)	Knowing antimicrobial resistance in practice: a multi-country qualitative study with human and animal healthcare professionals.	A	Veterinarians resort to broad-spectrum antibiotics in animals due to the limited capacity and affordability of laboratory facilities for culture and sensitivity testing.
2. (Adekanye *et al*., 2020).	Knowledge, Attitudes and Practices of Veterinarians Towards Antimicrobial Resistance and Stewardship in Nigeria.	A	Only 20% of respondents frequently conducted antimicrobial susceptibility testing (AST), citing the unavailability of veterinary lab services (82%) and owners' financial constraints (72%) as significant barriers.
3. (Achi *et al*., 2021)	Operationalising One Health in Nigeria: Reflections from a High-Level Expert Panel Discussion Commemorating the 2020 World Antibiotics Awareness Week.	A	Government veterinary laboratories are insufficient and where present, face low patronage due to cost and lack of awareness about the importance of testing.
4. (Ndahi *et al*., 2023)	Determination of antimicrobial use in commercial poultry farms in Plateau and Oyo States, Nigeria.	A	Farmers in the survey cited long distances (36%) and the high cost of analysis (30%) as the major challenges in utilizing laboratories.
**Lack of regulation and regulatory enforcement**			
1. (Omeiza *et al*., 2012)	Response of Nigerian farmers to a questionnaire on chloramphenicol application in commercial layers.	A	Only 26.7% of respondent farmers were aware that chloramphenicol was prohibited for use in food animals. 20.0% administered chloramphenicol in the form of veterinary preparation, while 14.6% administered the human preparation of the drug to poultry.

2. (Mbodi *et al*., 2014)	Determination of chloramphenicol residues in commercial chicken eggs in the Federal Capital Territory, Abuja, Nigeria.	A	66.6% of respondents (veterinarians) were unaware of the ban of Chloramphenicol in animal use, stated in Nigeria's Foods and Drugs Decre
3. (NCDC, 2017)	Antimicrobial Use and Resistance in Nigeria Situation Analysis and Recommendations.	A&H	Regulations to curb antimicrobial overuse, particularly concerning the sale of Prescription Only Medications over the counter (OTC), are loosely enforced.
4. (Ojo *et al*., 2017).	Activities and influence of veterinary drug marketers on antimicrobial usage in livestock production in Oyo and Kaduna States, Nigeria	A	Only 37% of the total marketers in both States knew about the existence of agencies regulating drug marketing activities and sales of antimicrobial agents.
5. (Alhaji & Isola, 2018)	Antimicrobial usage by pastoralists in food animals in North-central Nigeria: The associated socio-cultural drivers for antimicrobials misuse and public health implications.	A	Weak enforcement of laws regulating antimicrobial usage, significantly ($p<0.05$) influenced the misuse of antimicrobials on livestock by pastoralists
6. (Alhaji *et al*., 2019)	Survey on antimicrobial usage in local dairy cows in North-central Nigeria: Drivers for misuse and public health threats.	A	Non-enforcement of laws regulating AMU was found to significantly influence ($p<0.001$) antimicrobial misuse in cows by farmers.
7. (Ogwuche *et al*., 2021)	Antibiotic use practices of veterinarians and para-veterinarians and the implications for antibiotic stewardship in Nigeria.	A	56.5% of animal health professionals (veterinarians and para-veterinarians) in the survey were unaware of regulatory guidelines on antibiotic use in Nigeria.
8. (Bamidele *et al*., 2022)	Antimicrobial Usage in Smallholder Poultry Production in Nigeria.	A	Most farmers (96.2%) lacked awareness of regulations for antimicrobial usage and had no knowledge (98.2%) of the governing body overseeing the regulation of antimicrobials in livestock production
9. (Oloso *et al*., 2022)	Knowledge, Attitudes, and Risk Perception of Broiler Grow-Out Farmers on Antimicrobial Use and Resistance in Oyo State, Nigeria.	A	None of the participants (farmers) in the survey knew about any specific regulation or law in Nigeria controlling antimicrobial usage in broiler production.
**Poor regulation of pharmaceutical industry**			

1. (Adesokan *et al*., 2015)	Pattern of antimicrobial usage in livestock animals in south-western Nigeria: The need for alternative plans.	A	Access to veterinary drugs is unregulated and farmers acquire and administer drugs without the need for a veterinary prescription or supervision.
2. (NCDC, 2017)	Antimicrobial Use and Resistance in Nigeria Situation Analysis and Recommendations.	A&H	The Nigerian pharmaceutical sector is deemed chaotic, marked by counterfeit medicines, weak enforcement, and poor cooperation among law enforcement agencies.
3. (Fadare *et al*., 2018)	Drug promotional activities in Nigeria: impact on the prescribing patterns and practices of medical practitioners and the implications	H	64.8% of physicians agreed on a need to regulate the relationship between medical doctors and the pharmaceutical industry. 61% of physicians felt motivated to prescribe the drug promoted to them and food was the most common form of incentive (70.5%) given to physicians during promotional activities.
4. (Obasanya *et al*., 2022)	An appraisal of the contextual drivers of successful antimicrobial stewardship implementation in Nigerian health care facilities.	H	Participants observed that manufacturers' representatives are directly involved in supplying medicines to health facilities, making this alliance unwholesome and negatively impacting efforts to institutionalize antimicrobial stewardship programs.
**Prescribers marketing antimicrobials**			
1. (Ojo *et al*., 2017).	Activities and influence of veterinary drug marketers on antimicrobial usage in livestock production in Oyo and Kaduna States, Nigeria	A	Majority (85.3%) of the marketers who recommended antimicrobial agents to farmers were not licensed to prescribe antimicrobials for animal use and had no formal training on antimicrobial usage.
2. (Ogwuche *et al*., 2021)	Antibiotic use practices of veterinarians and para-veterinarians and the implications for antibiotic stewardship in Nigeria.	A	Veterinarians often sell antibiotics from their ambulatory drug stock, drug shop or pharmacy.
**Lack of access to veterinary expertise**			
1. (NCDC, 2017)	Antimicrobial Use and Resistance in Nigeria Situation Analysis and Recommendations.	A	7,688 veterinarians are registered with the Veterinary Council of Nigeria, and only about 2,000 in the public sector.
2. (Okpara *et al*., 2018)	Antimicrobial usage and presence of extended-spectrum β-lactamase-producing	A	51.5% of households had never sought veterinary services, and 23.1% had limited access to veterinary services.

	Enterobacteriaceae in animal-rearing households of selected rural and peri-urban communities.		
3. (Alhaji *et al*., 2019)	Survey on antimicrobial usage in local dairy cows in North-central Nigeria: Drivers for misuse and public health threats.	A	Grossly inadequate veterinary services associated with hard-to-reach terrains of Fulani pastoral communities influenced inappropriate use of antimicrobials
4. (Pearson & Chandler, 2019)	Knowing antimicrobial resistance in practice: a multi-country qualitative study with human and animal healthcare professionals.	A	Antimicrobials are frequently utilized as vehicles of care to accommodate the shortage of animal health professionals.
**Drug companies lobbying farmers**			
1. (Pearson & Chandler, 2019)	Knowing antimicrobial resistance in practice: a multi-country qualitative study with human and animal healthcare professionals.	A&H	Health professionals engaged with drug company representatives. These interactions significantly influenced prescription practices, as representatives prioritized business over patient care or the optimal use of medicines
**Lack of withdrawal period observation**			
1. (Omeiza *et al*., 2012)	Assessment of antimicrobial drug residues in beef in Abuja, the Federal Capital Territory, Nigeria.	A	53.2% of farmers administered drugs to cattle designated for meat, 24 hours prior to slaughter and many others 26.4% at 48 h prior to slaughter.
2. (Mbodi *et al*., 2014)	Determination of chloramphenicol residues in commercial chicken eggs in the Federal Capital Territory, Abuja, Nigeria.	A	12.9% and 6.2% of egg samples from poultry farms and market respectively, tested positive for chloramphenicol residues.
3. (Njoga *et al*., 2018)	Assessment of antimicrobial drug administration and antimicrobial residues in food animals in Enugu State, Nigeria.	A	65% of farms surveyed failed to observe withdrawal period in food animals.
4. (Alhaji *et al*., 2019)	Survey on antimicrobial usage in local dairy cows in North-central Nigeria: Drivers for misuse and public health threats.	A	81.3% of pastoralists failed to comply with withdrawal periods of drugs in cattle

5. (Oyedeji *et al*., 2019)	Determination of antibiotic residues in frozen poultry by a solid-phase dispersion method using liquid chromatography-triple quadrupole mass spectrometry	A	Nineteen different antibiotic residues were detected in imported frozen poultry. Sulfixosazole with a percentage violation of 80% in turkey gizzard, while sulfamethoxazole in chicken muscle had highest maximum concentration and 100% violation. This finding indicates a gap in regulation for antimicrobial residue screening, extending to imported products.
6. (Al-Mustapha *et al*., 2020).	Risk Perceptions of Antibiotic Usage and Resistance: A Cross-Sectional Survey of Poultry Farmers in Kwara State, Nigeria.	A	89.6% of farmers claim to observe the withdrawal period as stipulated on antibiotic sachet or vial, but none of them discarded the eggs in the course of antibiotic therapy
7. (Ojo *et al*., 2020).	Antimicrobial use and detection of cefotaxime-resistant Enterobacteriaceae in the pig production chain, Ogun State, Nigeria.	A	88.3% of farmers were aware of the importance of observing a withdrawal period before slaughter of pig, but only 26.7% observed it
8. (Achi *et al*., 2021)	Operationalising One Health in Nigeria: Reflections from a High-Level Expert Panel Discussion Commemorating the 2020 World Antibiotics Awareness Week.	A	Farmers often do not understand the need to observe the withdrawal periods of medicines before the slaughter of the animals, or where they do, sometimes fail to observe them.
9. (Alhaji *et al*., 2021)	Antimicrobial use, residue and resistance dissemination in freshwater fish farms of north-central Nigeria: One health implications.	A	94.9% of fish farmers reported non-compliance with withdrawal periods after AMU in farms
10. (Njoga *et al*., 2021)	Antimicrobial drug usage pattern in poultry farms in Nigeria: Implications for food safety, public health and poultry disease management.	A	62.3% of the poultry farmers who administered antimicrobials in the survey did not observe the recommended withdrawal period.
11. (Okocha *et al*., 2021)	Aquaculture management practices associated with antimicrobial residues in Southwestern Nigeria.	A	72.6% of farmers surveyed did not observe withdrawal period used in aquaculture.
12. (Olasoju *et al*., 2021)	Knowledge and practice of cattle handlers on antibiotic residues in meat and milk in Kwara State, Northcentral Nigeria.	A	52.7% of the cattle handlers in Kwara State had poor Knowledge and practices regarding antibiotic residues in meat and milk.

13. (Alhaji *et al*., 2023)	Antimicrobial usage and associated residues and resistance emergence in smallholder beef cattle production systems in Nigeria: A One Health challenge.	A	72.4% and 83.9% of the farmers on intensive and semi-intensive systems failed to observe withdrawal periods after antimicrobial use.
14. (Ndahi *et al*., 2023)	Determination of antimicrobial use in commercial poultry farms in Plateau and Oyo States, Nigeria.	A	74% of poultry farmers surveyed read about withdrawal period from the antibiotic containers/sachets, y*et al*l farmers sold eggs from birds under antibiotic treatment, as there was no provision for compensation for lost eggs.
**Poor formulation/combination of antimicrobials**			
1. (Akidarju *et al*., 2010)	An assessment of some poultry management practices and disease recognition by poultry farmers in Maiduguri arid zone, Nigeria.	A	Most farmers (72.3%) surveyed in Maiduguri, used multiple antibiotics in poultry for chemoprophylaxis.
2. (Oloso *et al*., 2019)	Knowledge, Attitudes, and Risk Perception of Broiler Grow-Out Farmers on Antimicrobial Use and Resistance in Oyo State, Nigeria.	A	Antimicrobials are packaged as fortified com-biotics (mixture of multiple antimicrobial agents, minerals, and vitamins, often included in sub-minimal levels or concentrations) majorly to serve the interest of growth promotion and production.
3. (Jibril *et al*., 2021)	Association between antimicrobial usage and resistance in Salmonella from poultry farms in Nigeria.	A	Several antimicrobial products surveyed from poultry contained two or more antimicrobials. Majority of the products contained compounds that are classified as critically important for human medicine such as erythromycin, aminoglycosides, oxazolidone and penicillin. And some were categorised as "Highest priority critically important" namely colistin and ciprofloxacin as listed by the WHO.
4. (Bamidele *et al*., 2022)	Antimicrobial Usage in Smallholder Poultry Production in Nigeria.	A	35.4% of poultry farmers used a combination of both traditional and western medications, in disease treatment and health management of poultry.
5. (Ndahi *et al*., 2023)	Determination of antimicrobial use in commercial poultry farms in Plateau and Oyo States, Nigeria.	A	Poultry farmers used cocktail of antibiotics, having up to six different classes with very high concentration of active ingredients. Some farmers used products containing a mixture of antibiotics and probiotics.

**Lack of funding for relevant research to back the need for AMR stewardship**			
1. (NCDC, 2017)	Antimicrobial Use and Resistance in Nigeria Situation Analysis and Recommendations.	A&H	There is absence of national coordination and reporting systems on antimicrobial use due to inadequate funding.
2. (NCDC, 2019)	One Health Strategic Plan Federal Republic of Nigeria.	A&H	There is limited government funding to tackle AMR.
3. (FGN NAP, 2017)	National Action Plan for Antimicrobial Resistance-Nigeria.	A&H	Insufficient funding limits AMR research and interventions in Nigeria.
**Hard to change practices & perceptions**			
1. (Nwolisa *et al*., 2006)	Prescribing Practices of Doctors Attending to Under Fives in a Children's Outpatient Clinic in Owerri, Nigeria.	H	A relatively high rate of poly pharmacy was observed among paediatricians attending to under five years. Antibiotics prescription accounted for 53.7% of prescribed medications by physicians.
2. (Esimone *et al*., 2007)	Utilization of antimicrobial agents with and without prescription by out-patients in selected pharmacies in South-eastern Nigeria.	H	Self-medication with antimicrobial is prevalent among outpatients and it is majorly driven by inadequate income to pay for the services, limited consultation, inadequate health facilities, impatience on the part of patients, and ignorance.
3. (Alex *et al*., 2019)	Knowledge of antibiotic use and resistance among students of a medical school in Nigeria.	H	20% of medical students surveyed, reportedly kept leftover pills, in case they might need it later
4. (Abubakar, 2020)	Antibiotic use among hospitalized patients in northern Nigeria: a multicenter point-prevalence survey.	H	Over 80% of hospitalised patients in the study, received at least one prescription for antibiotics. The prevalence of antibiotic use ranged from 72.9% in obstetrics and gynaecology to 94.6% in paediatric medical specialty.
5. (Chukwu *et al*., 2020)	A national survey of public awareness of antimicrobial resistance in Nigeria.	H	76.6% of the public surveyed felt powerless to stop the spread of AMR. 26.1% respondents admitted that they would stop taking antibiotics when they feel better as opposed to when they have completed the required dosage. 26.6% would use leftover drugs from a previous illness to treat a current illness

6. (Achi *et al*., 2021)	Operationalising One Health in Nigeria: Reflections from a High-Level Expert Panel Discussion Commemorating the 2020 World Antibiotics Awareness Week.	H	Many patients fear their infections will worsen without immediate antimicrobial treatment, leading to healthcare workers prescribing antimicrobials to maintain clientele and increase hospital income.
7. (Chukwu *et al*., 2021)	Antimicrobial resistance awareness and antibiotic prescribing behavior among healthcare workers in Nigeria: a national survey.	H	50.3% of health professionals reported that their prescribing behaviour could promote the development of antimicrobial resistance. However, several health professionals prescribed antibiotics for common viral infections such as sore throats (75.7%), measles (37.7%), common cold and flu (21.2%). Over 60.3% admitted prescribing antibiotics just to be on the safe side.
8. (Akande-Sholabi & Oyesiji, 2023)	Antimicrobial stewardship: knowledge, perceptions, and factors associated with antibiotics misuse among consumer's visiting the community pharmacies in a Nigeria Southwestern State	H	Factors associated with antibiotics use, cited by participants in the study included delay in obtaining test reports from the laboratory (47.1%), antibiotics can offer quicker relief from symptoms (75.8%). 77.4% claimed that their usage was based on past prescriptions from doctors or other healthcare professionals, while 73.7% expressed comfort in using antibiotics after registering complaints with pharmacists. 47.7% acknowledged the convenience of nearby pharmacies in acquiring antibiotics and 32.2% of the respondents disclosed having a phobia for injections. 33.5% expressed a preference for antibiotics over expensive consultation fees, and 13.8% admitted to occasionally combining antibiotics with herbal preparations
9. (Isah et al., 2023)	Assessment of public knowledge and attitude toward antibiotics use and resistance: a community pharmacy-based survey	H	More than half of the respondents (58.3%) specified that antibiotics are used immediately after unprotected sexual intercourse to prevent sexually transmitted diseases. 50.9% reported that antibiotics could be used to relieve fever and pain. 30% of the respondents affirm that they expect antibiotics to be prescribed with an antimalarial, 22.1% construed that antibiotics cure their cold/sore throat faster, while 27.9% reported that

			using leftover antibiotics was permissible when they had cold or similar symptoms.
10. (Amorha et al., 2024)	Assessment of Community Pharmacists' Knowledge, Perception and Practice Regarding Antimicrobial Stewardship in Ebonyi State, Southeastern Nigeria	H	40% of the respondents (community pharmacists) reported that they dispense antibiotics without prescription often/always.
11. (Nwafia et al., 2024)	Point Prevalence Survey of Antimicrobial Prescription and Consumption in a Nigerian Tertiary Hospital: A Gateway to the Antimicrobial Stewardship Program	H	94.9% of antimicrobial treatments were on empirical basis undermining the benefits of the use of the microbiology laboratory in guiding the therapy
12. (Sulaiman et al., 2024)	An assessment of the index of rational drug prescribing for severe acute respiratory infections among hospitalised children in Northern Nigeria: a retrospective study	H	Study showed irrational antibiotic prescription among hospitalized children diagnosed with severe acute respiratory infections, with 99.7% of patients receiving at least an antibiotic prescription.
13. (Popoola et al., 2024)	A national survey of the antibiotic use, self-medication practices, and knowledge of antibiotic resistance among graduates of tertiary institutions in Nigeria.	H	47.7 % of respondents self-medicate with antibiotics, 54.9 % of respondents don't carry out laboratory investigations before using antibiotics, and 70.1 % reduce or stop taking antibiotics on feeling better.
**Lack of baseline information on AMR**			
1. (NCDC, 2017)	Antimicrobial Use and Resistance in Nigeria Situation Analysis and Recommendations.	Not specified	Institution-based AMR surveillance often lacks national oversight and coverage in the country. There is limited information regarding the burden of resistance in Nigeria, resistance primarily through the lens of clinical medicine rather than an ecological problem.
2. (Chukwu et al., 2024)	Implementation of antimicrobial stewardship programs: A study of prescribers' perspective of facilitators and barriers	H	40% of participating clinicians & pharmacists believed that there was paucity of data on AMR, resulting in difficulty to access the true situation, address appropriately and proffer strategies to ameliorate future AMR incidents from occurring.
**Disconnect between Federal and State level**			
1. (FGN NAP, 2017)	National Action Plan for Antimicrobial Resistance-Nigeria.	H	The National Health Policy assigns healthcare responsibilities to different government levels to create a coordinated health

			system. However, this autonomy leads to varying priorities, policies, and resource allocation

AMR DRIVERS

AUTHOR/YEAR	TITLE	SECTOR	FINDINGS/OBSERVATIONS
**Lack of IPC**			
1. (NCDC, 2017)	Antimicrobial Use and Resistance in Nigeria Situation Analysis and Recommendations.	H	Infection prevention and control (IPC) has been a neglected area in many health care facilities despite many policies related to IPC programmes in Nigeria.
2. (Ijarotimi *et al*., 2018)	Knowledge of Lassa fever and use of infection prevention and control facilities among health care workers during Lassa fever outbreak in Ondo State, Nigeria.	H	Only 23.2% of respondents had received IPC training and none of the health facility had all Infection Prevention and Control (IPC) requirements.
3. (Adekanye *et al*., 2020).	Knowledge, Attitudes and Practices of Veterinarians Towards Antimicrobial Resistance and Stewardship in Nigeria.	A	51% of the participants (veterinarians) in the study expressed the view that using prophylactic antibiotics was justified in situations where farm biosecurity was poor.
4. (Nwaokenye *et al*., 2020)	Perceptions of Nigerian healthcare workers towards hand hygiene: a qualitative study.	H	Lack of resources, absence of regulations and poor working conditions were impediments to the successful implementation of good hand hygiene practices.
5. (Onyedibe *et al*., 2020)	Assessment of hand hygiene facilities and staff compliance in a large tertiary health care facility in northern Nigeria: a cross sectional study.	H	Poor hand hygiene compliance (31%) was reported among healthcare workers in the study. Hand hygiene facilities were deficient as 87% of the units did not have alcohol-based hand rubs; only 28% had uninterrupted tap water, while 72% utilized cup and bucket; and 58% had no hand drying facilities.
6. (Ilesanmi *et al*., 2021)	Infection prevention and control during COVID-19 pandemic: realities from health care workers in a north central state in Nigeria.	H	Lack of IPC infrastructures in healthcare settings, inadequate supply of personal protective equipment (PPE), poor political will and inadequate management support were highlighted as challenges to IPC implementation.

7. (Lowe *et al*., 2021)	Challenges and opportunities for infection prevention and control in hospitals in conflict-affected settings: a qualitative study.	H	Inadequate hospital infrastructure, resource and workforce shortages, education of staff, inadequate in-service IPC training and supervision, and large visitor numbers, were identified as barriers to IPC in health facilities.
8. (Aika & Enato, 2022)	Health care systems administrators perspectives on antimicrobial stewardship and infection prevention and control programs across three healthcare levels: a qualitative study.	H	Common IPC challenges observed across the healthcare institutions in the study include inadequate waste disposal, lack of personal protective equipment (PPEs) and behavioural change among healthcare providers. Limited supply of waste disposal materials and PPEs were cited in all facilities as a major IPC challenge. Formal IPC programme was reported in tertiary health institutions. In contrast, neither secondary nor primary facilities have established formal IPC programme in place.
9. (Falana et al., 2024)	Barriers to infection prevention and control implementation in selected healthcare facilities in Nigeria	H	Inadequate infection prevention and control materials, poor waste management, non-compliance of patients with infection prevention and control protocols, and poor infrastructure were identified as major barriers to infection prevention and control implementation.
**Spillover from pharmaceutical wastes**			
1. (Lateef, 2004)	The microbiology of a pharmaceutical effluent and its public health implications.	Not specified	Microbiological evaluation of pharmaceutical effluents revealed that 80% of bacterial isolates from a pharmaceutical effluent were resistant to amoxycillin, 76% to nitrofurantoin, 64% to cotrimoxazole and augmentin, 60% were resistant to nalidixic acid, 52% were resistant to tetracycline and ofloxacin, while resistance of 12% was obtained for gentamicin.
2. (Lateef *et al*., 2007)	Bacteriology and genotoxicity of some pharmaceutical wastewaters in Nigeria.	Not specified	Bacterial isolates from pharmaceutical effluents exhibited patterns of multiple drug resistance ranged from 10% for gentamicin to 100% for augmentin, amoxycillin, cloxacillin and nalidixic acid.

3. (Ngwuluka *et al*., 2011)	An assessment of pharmaceutical waste management in some Nigerian pharmaceutical industries.	H	79.4% of Nigerian pharmaceutical companies in a survey, failed to remove pharmaceuticals from wastewater discharged into the environment.
4. (NCDC, 2017)	Antimicrobial Use and Resistance in Nigeria Situation Analysis and Recommendations.	A&H	Inadequate treatment of wastewater from drug manufacturers, and effluents from agriculture and aquaculture are major drivers of resistance in the environment. However, not much is known about burden of resistance in the Nigerian environment, as AMR is still viewed largely from the lens of clinical medicine and not as an ecological problem.
5. (Ebele *et al*., 2020)	Occurrence, seasonal variation and human exposure to pharmaceuticals and personal care products in surface water, groundwater and drinking water in Lagos State, Nigeria.	Not specified	Amoxicillin was found to be the most prevalent compound detected in sampled water bodies in Lagos state. Its median concentrations were 1614 ng/L in surface water, 238 ng/L in ground water, and 358 ng/L in drinking water.
6. (Ogunbanwo *et al*., 2022)	High Concentrations of Pharmaceuticals in a Nigerian River Catchment.	Not specified	High concentrations of pharmaceuticals were detected in river catchment in Lagos state, with sulfamethoxazole having the highest concentration 129 μg/ L. Sewage effluent was found to be more important than drug manufacturing waste in polluting rivers in this study.
**Unsanitary processes and inadequate hygiene in abattoirs**			
1. (Adelowo *et al*., 2012)	Environmental impact assessment of Attenda abattoir, Ogbomoso southwestern Nigeria on surface and groundwater quality using geo-electrical imaging and microbiological analysis.	A	Study showed contaminated wastewater, containing clinically relevant antimicrobial drug resistant pathogens are discharged from abattoirs into water bodies
2. (Ikegbunam *et al*., 2014)	Abattoirs as Non-Hospital Source of Extended Spectrum Beta Lactamase Producers: Confirmed by the Double Disc Synergy Test and Characterized by Matrix-Assisted Laser Desorption/Ionization Time of Flight Mass Spectrometry.	A	Study established the presence of Extended Spectrum Beta Lactamase (ESBL) producing bacteria in the gut of animals from abattoirs and tables of meat sellers, at a comparable rate to hospital intensive care units. Study highlights the need for improved hygiene in abattoir among buyers and sellers.

3. (Agbaje *et al*., 2019)	Antimicrobial resistance profiles of Salmonella serovars isolated from dressed chicken meat at slaughter in Kaduna, Nigeria.	A	Study showed high contamination of potentially zoonotic non-typhoidal Salmonella serovars in poultry meat. 85.7% Salmonella isolates from this study displayed antimicrobial resistance to ciprofloxacin, followed by nalidixic acid (75%), sulfamethoxazole (67.8%) and tetracycline (89.3%). Meat contamination with multi-drug resistant Salmonella serovars was attributed to unhygienic processes such as lack of proper separation of dirty and clean areas during evisceration in the slaughterhouse, unhygienic processors and equipment during processing.
4. (Egbule *et al*., 2020)	Beta-Lactamase-Producing Escherichia coli Isolates Recovered from Pig Handlers in Retail Shops and Abattoirs in Selected Localities in Southern Nigeria: Implications for Public Health.	A	Study showed high prevalence of extended-spectrum beta-lactamase-producing *Escherichia coli* in abattoirs and retail shops, attributed to poor hygiene practices and handling.
5. (Okafor *et al*., 2020)	Occurrence of multidrug-resistant Salmonella in cattle carcass and contact surfaces in Kwata slaughterhouse, Awka, Anambra State, Nigeria.	A	Study revealed antimicrobial-resistant Salmonella strains were isolated from beef and slaughter/processing facilities. This is indicative of poor hygiene and processing in the slaughterhouse. Study observed inadequate waste disposal and disinfection of slaughter floor and processing facilities.
6. (Shaibu *et al*., 2021)	Isolation and antibiogram of Salmonella species from slaughtered cattle and the processing environment in Abuja abattoirs, Nigeria.	A	Study detected the presence of multi-drug resistant Salmonella species in beef and the processing environment at abattoirs in Abuja, majorly attributed to insufficient hygienic practices in the studied abattoirs.
**Environmental degradation**			
1. (Paul *et al*., 2012)	Local poultry biosecurity risks to highly pathogenic avian influenza in Kaduna State, Nigeria	A	Haphazard manure disposal, especially from poultry, is a major source of environmental degradation in Nigeria
2. (NCDC, 2017)	Antimicrobial Use and Resistance in Nigeria Situation Analysis and Recommendations.		The environmental impact of AMR is poorly understood in Nigeria, as it is mainly viewed as a medical problem, rather than an ecological problem.

3. (Gbotosho & Burt, 2013)	Environmental and health impacts of poultry manure disposal methods: A case study of Lagelu and Egbeda local government areas in Oyo State, Nigeria.		Indiscriminate disposal of manure is a major source of environmental degradation.
